# Supplementary material for: Crystal Structure of a Eukaryotic GEN1 Resolving Enzyme Bound to DNA
Source: Cell Rep. 2015 Dec 10;13(11):2565–75. doi: 10.1016/j.celrep.2015.11.042 (PMC4695337; doi:10.1016/j.celrep.2015.11.042)
Supplement: Document S1. Supplemental Experimental Procedures, Figures S1–S7, and Table S1 [file mmc1.pdf]

Cell Reports

Supplemental Information

Crystal Structure of a Eukaryotic GEN1 Resolving Enzyme Bound to DNA

Yijin Liu, Alasdair D.J. Freeman, Anne-Cécile Déclais, Timothy J. Wilson, Anton Gartner, and David M.J. Lilley

SUPPLEMENTARY FIGURES

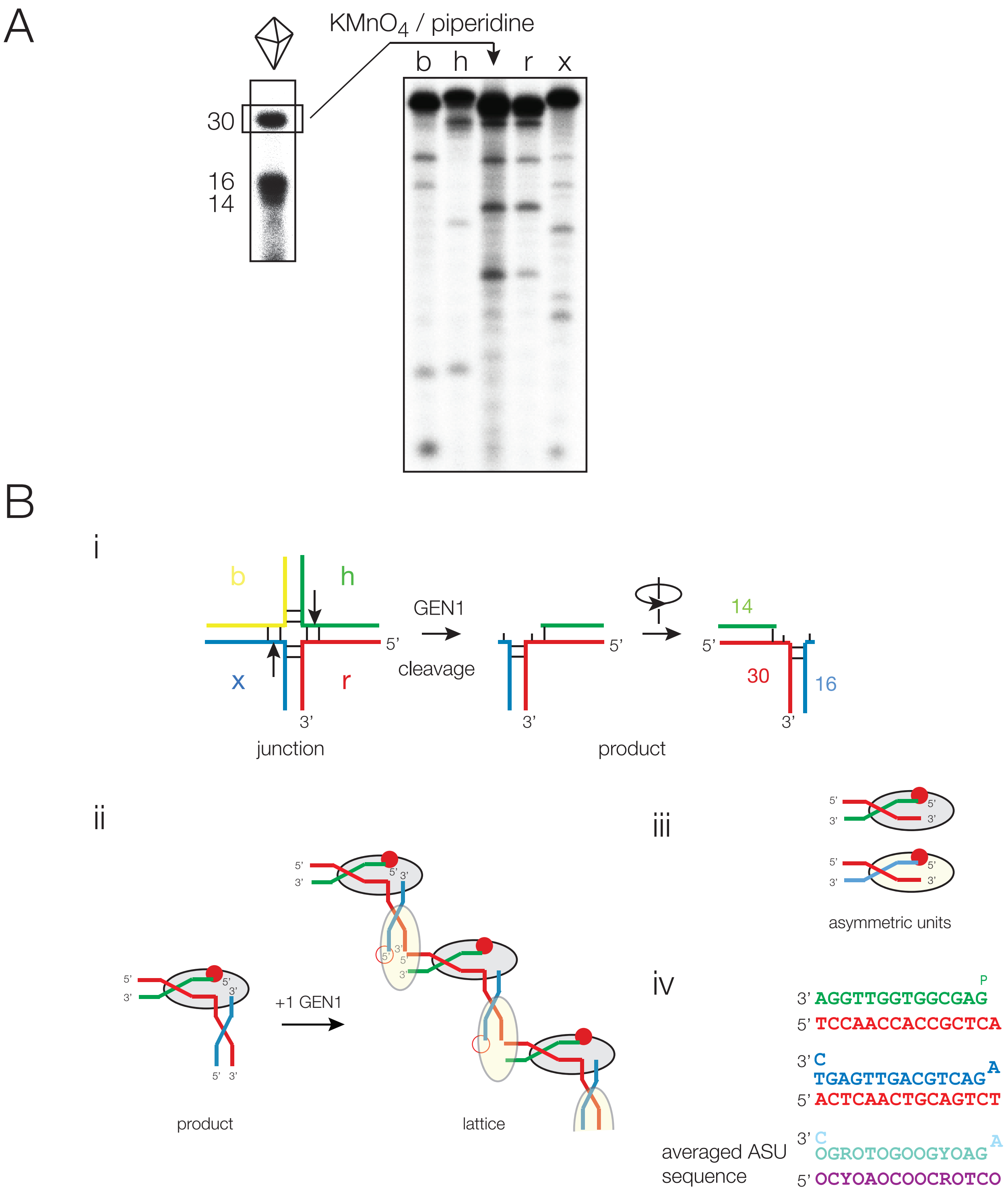

Figure S1. The Contents of the Crystal, and the Probable Origin of the Observed Lattice; Related to Figure 1.
A. Analysis of DNA in crystals used to collect X-ray diffraction data. Crystals previously used for data collection at the synchrotron were resuspended in 10 μl 10 mM Hepes (pH 7.5), 50 mM KCl, 1 mM $CaCl_2$ and incubated with 20 μg of proteinase K for 16 h at 37°C. The protease was inactivated by incubation with 1 mM PMSF for 20 min, and DNA denatured by heating. The DNA was radioactively [5'-^{32}P]-labeled using T4 polynucleotide kinase and [γ-^{32}P] ATP. The DNA was analyzed by electrophoresis in a 20% (19:1) polyacrylamide in the presence of 8M urea (*left*). After phosphorimaging, two bands of radioactive DNA are seen. The upper one corresponds to 30 nt, while the lower one is

interpreted in terms of the presence of two species of 16 and 14 nt. DNA was recovered from the upper band by electroelution. This was reacted with $KMnO_4$, and followed by incubation with piperidine to cleave the backbone at the positions of modified thymine nucleotides as detailed in the Material and Methods. Each strand of the junction used in crystallization was also independently labeled and subjected to $KMnO_4$ / piperidine reaction. All five samples were electrophoresed in a 20% (19:1) polyacrylamide gel in buffer containing 8M urea (*right*). It is clear that the mobility of the full-length strand and the pattern of bands of greater mobility (corresponding to cleavage at permanganate-modified thymine nucleotides) observed in the 30 nt oligonucleotide extracted from the crystal closely matches that of the r strand. By contrast, bands unique to the b strand are not visible in this gel. Tracks L to R : b strand, h strand, extracted 30 nt oligonucleotide, r strand and x strand.

B. The probable origin of the CtGEN1-DNA complexes observed in the crystal. All available evidence indicates that the crystal contains a complex of a monomer of CtGEN1 with the product of resolution cleavage that predominantly contains the r strand.

i) The major cleavage of the four-way junction (with four arms each of 15 bp, shown left) by CtGEN1 occurs at the sites in the h and x strands shown by the arrows (Freeman et al., 2014), to produce the r-strand product (center). This is also shown rotated around the vertical axis (right). The overhanging nucleotides are not shown in the remaining diagrams.

ii) Schematic of the complex of the product DNA bound to one molecule of CtGEN1 (left). CtGEN1 is depicted as the grey ellipse, with the active site indicated by the red spot. Binding of a second molecule of GEN1 (colored yellow) in a quasi-equivalent manner with the free ends (i.e. the 3' end of the h strand and the 5' end of the x strand) generates the end to end chain of complexes observed in the crystal lattice. However the parallel chains that run through the crystal lattice (see supplementary movie S2) are not all in register; the products in one strand could align with those of an adjacent strand, or they could be offset such that the h strands in one chain align with the x strands of an adjacent strand. This is because all of the contacts within the lattice are protein mediated, and GEN1 binds each half of the product in an equivalent manner. This lack of alignment results in the averaging of the DNA sequences in the electron density map.

iii) This generates two kinds of complex as the asymmetric unit, each containing a different half of the product nicked duplex.

iv) The quasi-equivalence in the asymmetric unit generates an averaged nucleotide sequence. These sequences are written with the top strand running 3' to 5' in each case. The sequence of the DNA cannot be read from the electron density map in a straightforward way, but can be interpreted in terms of an average of the h and x sequences. R = purine, Y = pyrimidine, O = averaged purine/pyrimidine. The 3'-cytosine and 5'-adenosine of the x strand should have occupancies of 0.5, but are not observed. There is space for them to be present, but they are probably too mobile within the structure to be observable.

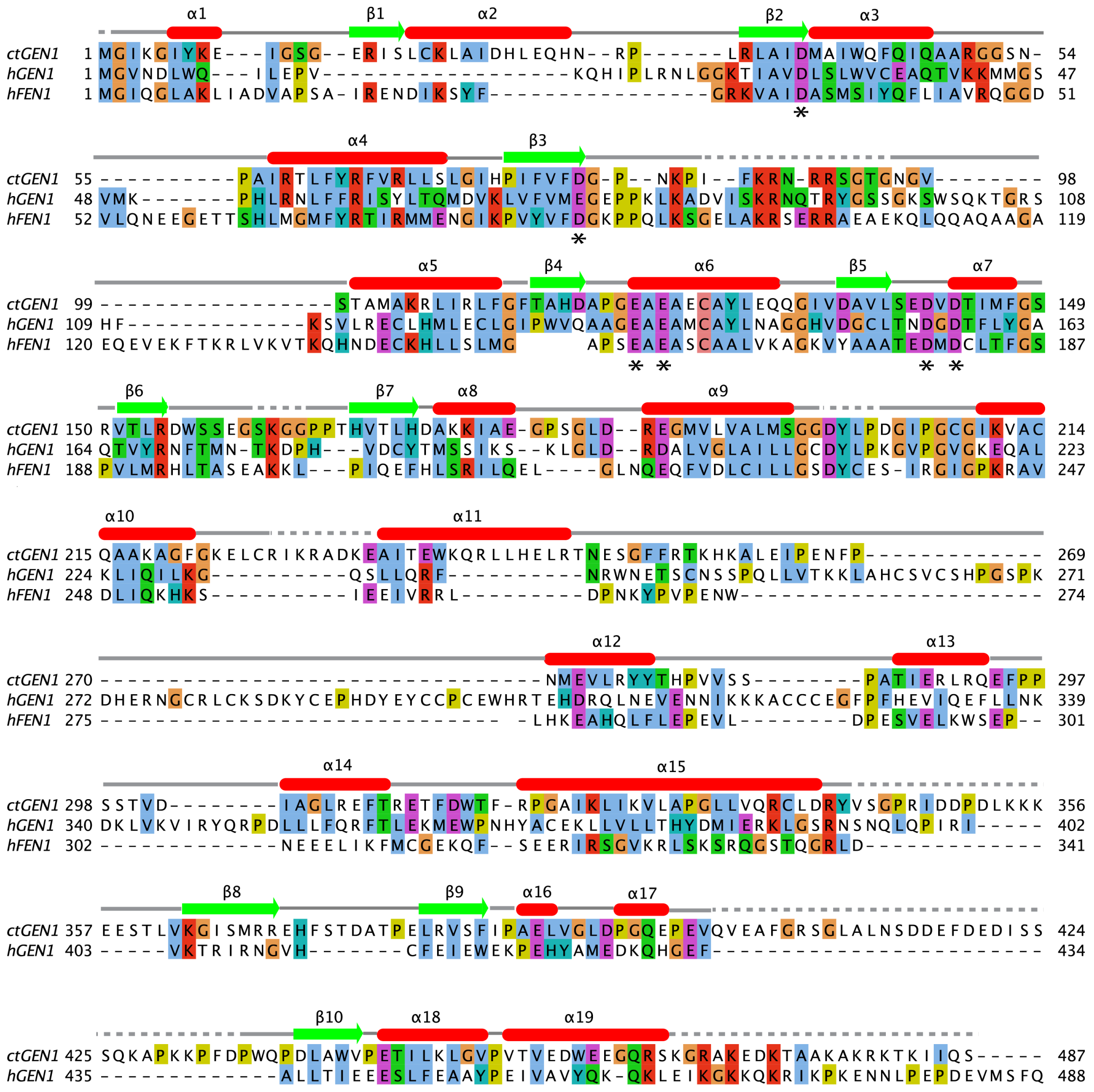

Figure S2. An Alignment of CtGEN1 (ctGEN1), Human GEN1 (hGEN1) and Human FEN1 (hFEN1) with the Secondary Structure Elements of CtGEN1; Related to Figure 2. Secondary structure is shown as α-helix (red) and β-sheet (green). Regions not observed in the electron density maps are shown by a broken line. Active site acidic residues are marked by an asterisk.

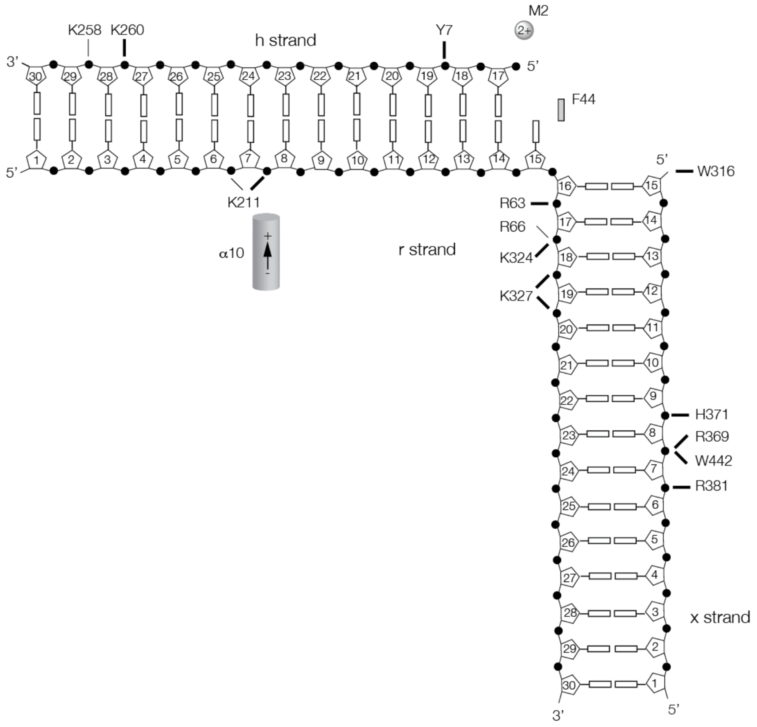

Figure S3. A Map of DNA-Protein Contacts in the Functional Unit of the CtGEN1 Complex; Related to Figure 3.
The cylinder shows the helix dipole of α10, with its N-terminus (positive pole) directed at a phosphate group in the cleaved DNA helix.

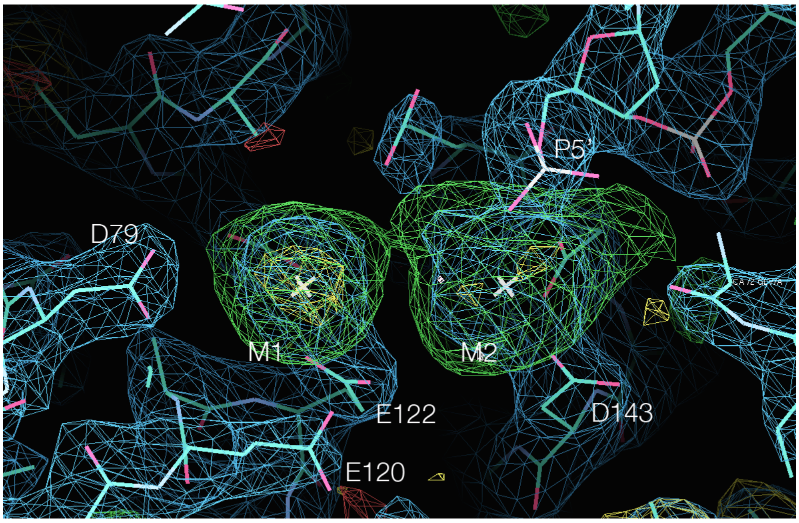

Figure S4. Electron Density Map of the Active Site for the Mn^{2+}-Soaked Crystals using Anomalous Dispersion by Mn^{2+} Ions; Related to Figure 4.
The data were collected at λ = 0.91 Å in the high energy remote area of the manganese K absorption edge to prevent protein crystal radiation damage. *Blue* contouring : 2$\mathbf{F_o}$-$\mathbf{F_c}$ map contoured at 1.5 σ. *Green* contouring : $\mathbf{F_o}$-$\mathbf{F_c}$ simulated annealing omit map contoured at 3 σ showing density consistent with the presence of two metal ions. *Yellow* contouring: Anomalous dispersion map for Mn^{2+} ions contoured at 3 σ. These data indicate that during the crystal soaking the Mg^{2+} ion is mostly retained in the M2 position, but the M1 site becomes predominantly occupied by a Mn^{2+} ion.

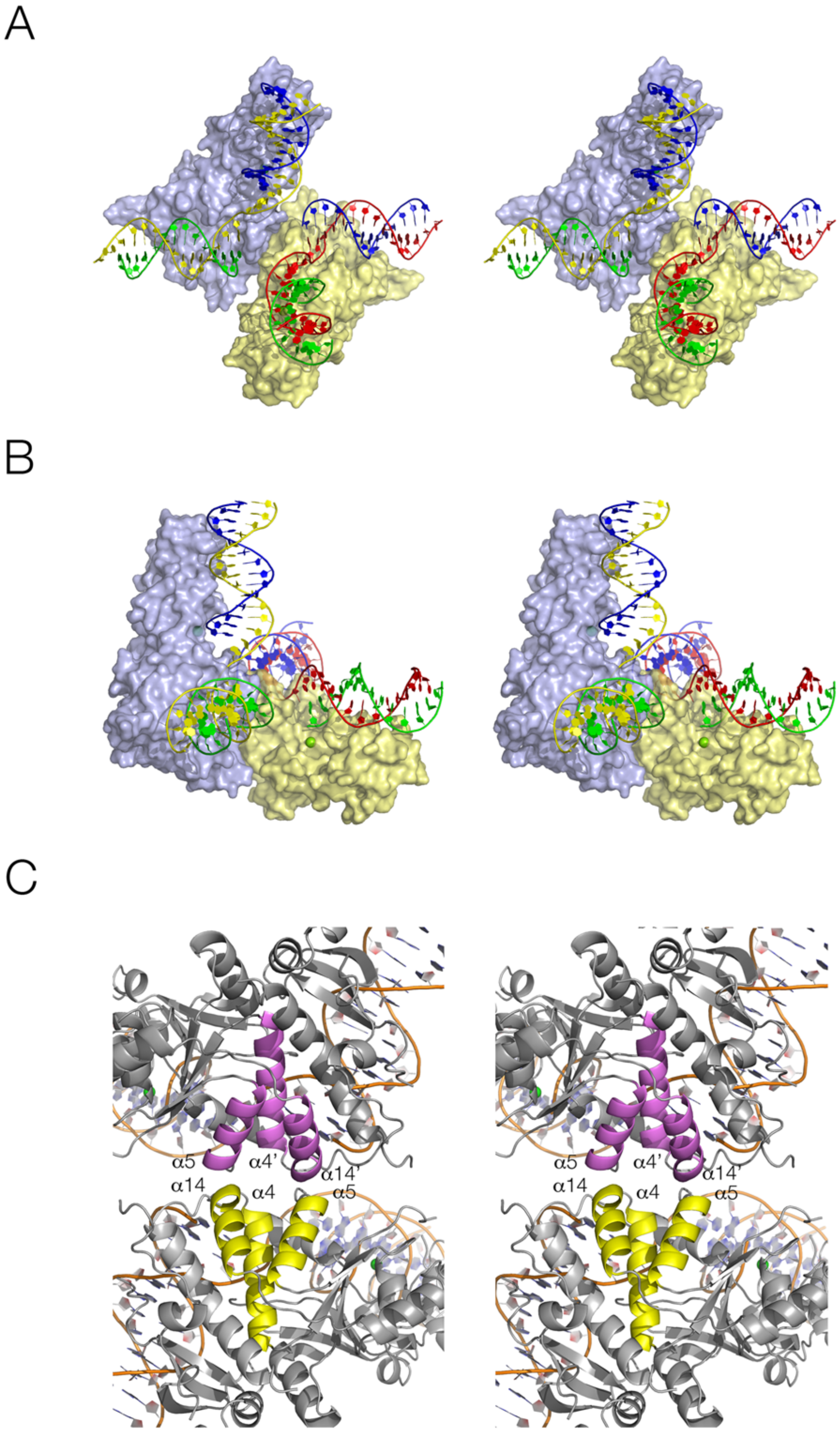

Figure S5. Parallel-Eye Stereoscopic Views of the Dimeric Form of the Complex Found in the Crystal Lattice; Related to Figure 5. **A, B**. Two views of the complex, with the strands colored to match the expected products of resolution as shown in the scheme in Figure S1Bi.

C. Close view of the dimerization interface, comprising helices α4, α5 and α14 from each monomer (highlighted yellow and magenta).

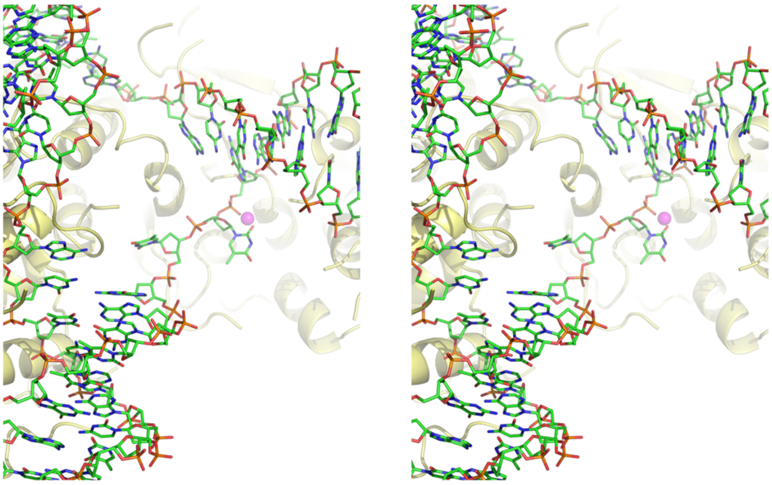

Figure S6. Close View of the Reconnected Junction; Related to Figure 5.
Parallel-eye stereoscopic close view of the sections that have been reconnected to form the fully-intact junction.

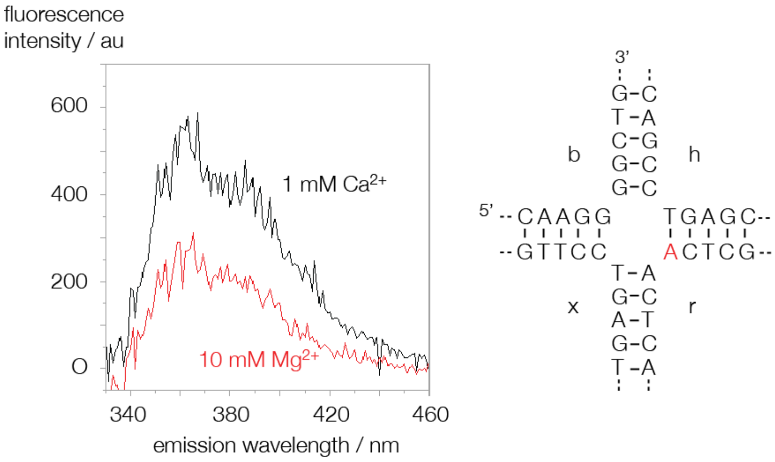

Figure S7. Fluorescence Spectra of 2-Aminopurine at the r-1 Position of the Junction as a Function Enzymatic Cleavage; Related to Figure 7.
Fluorescence emission spectra of junction 3 with adenine substituted by 2-aminopurine at the r-1 position (colored red) complexed with CtGEN as a function of the nature of the divalent cation. The spectrum was recorded in 1 mM Ca^{2+} ions (black), where the enzyme is inactive, and after the addition of 10 mM Mg^{2+} ions to allow enzymatic cleavage to occur.

SUPPLEMENTARY MOVIES

Movie S1. The Relationship between the Asymmetric Unit, the Functional Unit and the Lattice. Related to Figure 1.
Scene 1 : The single functional unit, corresponding to the product of GEN1 cleavage. A single GEN1 monomer (blue) binds two helices connected by the continuity of the red strand. The strands are colored as in Figure 1.
Scene 2 : Two more functional units are added, directly repeated head-to-tail , as found in the crystal lattice.
Scene 3 : Two additional GEN1 monomers (yellow) bind to connect the functional units. These then rotate to show how they are bound on opposite faces of the DNA helices.
Scene 4. The blue GEN1 monomers are hidden to show how the additional yellow monomers are bound in an equivalent manner to the first blue monomers. Within these complexes the two DNA helices are not connected by a central phosphate on the red strand.

Movie S2. The Presence of a Dimeric GEN1 Complex within the Crystal Lattice. Related to Figure 5.
Scene 1 : DNA molecules in the crystal lattice, beginning by viewing down the three-fold axis. The three linear chains of products (like that shown in Movie S1) are colored red, blue and green, and form parallel chains through the lattice. The lattice is then rotated sequentially about orthogonal axes.
Scene 2 : most of the molecules become grey, with just two product molecules left colored red and green. The view now zooms into these, and the remaining molecules fade out.
Scene 3 : The two DNA products juxtaposed in the lattice are rotated, and recolored to match the coloring of the strands of the product used in Figure 1A.
Scene 4 : Two bound CtGEN1 molecules now become visible, forming a dimeric complex interacting via a protein-protein interface.

Movie S3. The Dimer Complex, Closely Related to a Four-way Junction bound to two GEN1 Monomers. Related to Figure 5.
Scene 1 : The dimeric complex of CtGEN1 is shown in cartoon form. This rotates and switches to space-filling representation.
Scene 2 : The two bound DNA products now appear. Rotation occurs - note the view down the axis of the coaxial (uncleaved) arms as it passes, also showing that the remaining (cleaved) arms are mutually perpendicular.
Scene 3: One of the two products is recolored so that the four strands are now colored as in the complete junction (Figure 1A).
Scene 4. The strands become reconnected to form an intact junction. The view now zooms into the center of the junction, which is now shown with bonds in stick form. Notice that the reconnected green h strand is now close to the active site, indicated by the magenta metal ion. The strand reconnection was modeled manually – see text for details.

SUPPLEMENTARY TABLE S1

mutant	K_d / nM	rate / s^{-1}
WT	7.7 ± 0.01	0.12 ± 0.01
D38A	6.2 ± 0.05	0.01 ± 0.001
D79A	5.9 ± 0.05	$3.9 \times 10^{-6} \pm 7 \times 10^{-7}$
E120A	6.5 ± 0.10	$0.0012 \pm 7 \times 10^{-6}$
E122A	4.6 ± 0.04	$5.5 \times 10^{-6} \pm 3 \times 10^{-7}$
D141A	10.0 ± 0.01	$4.3 \times 10^{-4} \pm 3 \times 10^{-5}$
D143A	7.1 ± 0.01	0.001 ± 10^{-4}

Table S1. The Cleavage Activity and Binding Affinity of Point Mutants of CtGEN1; Related to Figure 4.
Affinities were measured by separation of free and complexed junction 3 by gel electrophoresis, and data fitted to the Hill equation. There was a spread of Hill coefficients between 5.3 (wild type CtGEN1) and 3.1. The rates of cleavage were measured under single-turnover conditions, and cleavage progress curves fitted to single exponential functions.

MATERIALS AND METHODS

Sample preparation and purification

A synthetic gene encoding *C. thermophilus* GEN1 1-487 with codon optimization for *E. coli* (Invitrogen) (Freeman et al., 2014) was inserted into a pET series plasmid construct that adds a six-histidine tag at the C-terminal end. The plasmid was transformed into *E. coli* BL21(DE3) RIL (Stratagene). A single colony was incubated in 1 l LB culture at 37°C for 8 h, protein expression induced by addition of IPTG at a final concentration of 0.3 mM, and incubated at 18°C for a further 20 h. Cells were harvested by centrifugation and re-suspended in 2x phosphate-buffered saline containing 10 mM imidazole and frozen at -20°C. After thawing at room temperature and sonication, lysed cells were centrifuged at 20,000g. The supernatant containing the recombinant protein was purified in sequential chromatographic steps using a Ni-NTA gravity column (HiTrap™), heparin HP, Superdex 75 10/300 GL gel filtration and Mono S 5/50 GL (GE healthcare) ion exchange. The purified protein was concentrated to ~100 µM in 5 mM Hepes (pH 7.0), 300 mM NaCl and stored on ice until used in crystallization trials. Purified CtGEN1 migrated as a single band on an overloaded polyacrylamide gel in the presence of SDS.

For crystallization, four strands of 30 nt were chemically synthesized using β-cyanoethyl phosphoramidite chemistry (Beaucage and Caruthers, 1981; Sinha et al., 1984). The oligonucleotides were not phosphorylated at their 5'-termini. Fully deprotected oligonucleotides were purified by gel electrophoresis in 15% (w/v) polyacrylamide gels in 90 mM Tris.borate (pH 8.5), 2 mM EDTA (TBE buffer) containing 8 M urea, and recovered by Elutrap (Whatman) electroelution and ethanol precipitation. The four strands were mixed in equimolar ratio in 5 mM Hepes (pH 7.0), 50 mM NaCl at 90°C and slowly cooled to 4°C. The hybridized junction was purified by electrophoresis in 10% polyacrylamide gels in non-denaturing conditions, and recovered by electroelution and ethanol precipitation. The purified junction was dissolved in 5 mM Hepes (pH 7.0), 50 mM NaCl at a concentration of 100 µM.

DNA sequences used for crystallization (all sequences are written 5' to 3') :
b strand : TCCGTCCTAGCAAGGGGCTGCTACCGGAGG
h strand : CCTCCGGTAGCAGCCTGAGCGGTGGTTGGA
r strand : TCCAACCACCGCTCAACTCAACTGCAGTCT
x strand : AGACTGCAGTTGAGTCCTTGCTAGGACGGA

Crystallization

Equal volumes of CtGEN1 protein (either native or selenomethione-substituted) and four-way DNA junction both at 100 μM were mixed together, and $MgCl_2$ added to a final concentration of 4 mM. This was then diluted with an equal volume of 2x crystallization buffer containing 100 mM Hepes (pH 7.5), 20% PEG10000 and 2 μl hanging drops suspended above crystallization buffer at 7°C in sealed wells. Crystals of dimension 100 x 100 x 100 µm grew in 2-3 weeks. They were attached to loops and soaked

in cryo-protectant containing 100 mM Hepes (pH 7.5), 20% PEG10000 and 30% sucrose. An additional 1 mM $MnCl_2$ was added for the Mn^{2+} complexes. The crystals were then dehydrated by vapor diffusion equilibration with saturated KNO_3 at 7°C for 2 h, and stored under liquid nitrogen.

Data collection and structure determination

Data sets for the native CtGEN1 complex in Mg^{2+} or Mn^{2+} ions were acquired at the Diamond synchrotron beamline I24 at 2.5 Å, and at the European Synchrotron Radiation Facility (ESRF), Grenoble at 2.6 Å resolution respectively. A single-wavelength anomalous dispersion (SAD) data set for selenomethonine-containing CtGEN1 complex was acquired at the Diamond synchrotron beamline I03 at 3.15 Å resolution. Data sets were checked by L-test, from which it was concluded that there was no twinning. Initial phases were acquired from the SAD data by locating the 8 selenium atoms with Autosol in the PHENIX suite (Adams et al., 2010). The initial model was generated automatically by PHENIX autobuild wizard, and then applied to the native data sets by molecular replacement using Phaser (McCoy et al., 2007). The model was then adjusted manually using Coot (Emsley et al., 2010), and subjected to several rounds of adjustment and optimization using Coot, REFMAC (Murshudov et al., 2011) and phenix.refine. To avoid any uncertainty arising from the space group determination, the original diffraction data sets were processed in the 21 possible space groups for macromolecules in hP (hexagonal Primitive) lattices. The final refined ctGEN1 protein structure (without DNA) was used as the model to search molecular replacement solutions for each space group using Phaser. We found that only P3121 and P3221 gave the correct solution with a Z-score of 12.7. None of the other space groups gave any solution with Z-score > 5.7. Molecular models are displayed and movies were prepared using PyMOL (DeLano, 2002).

	CtGEN1-SeMet	CtGEN1-Mg^{2+}	CtGEN1-Mn^{2+}
**Data collection**			
Wavelength (Å)	0.9796	0.9750	0.91376
Resolution range (Å)	32.43 - 3.15 (3.23 – 3.15)	69.26 - 2.51 (2.71 - 2.51)	49.29-2.60 (2.74 – 2.60)
Space group	P $3_1$21	P $3_1$21	P $3_1$21
Unit cell (Å, °)	99.08, 99.08,120.67<br>90, 90, 120	98.11, 98.11,119.58<br>90, 90, 120	98.58,98,58,119.62<br>90, 90, 120
Total reflections	241448 (18210)	132486 (27582)	391739 (59675)
Unique reflections	12299 (907)	23324 (4720)	21079(3037)
Multiplicity		5.6 (5.8)	
Anomalous Multiplicity	10.5 (10.5)		9.8 (10.2)
Completeness (%)	99.9 (100.00)	99.70 (100.00)	100 (100)
Mean I/s (I)	18.5 (1.7)	12.8 (1.8)	11.2 (2.0)
Wilson B-factor	100.753	42.53	69.46
R_{merge}	0.108 (1.976)	0.151 (0.764)	0.173 (1.683)
$CC_{1/2}$	0.999 (0.682)	0.991 (0.646)	0.999 (0.701)
Figure of merit	0.355		

Refinement

R-work	0.2232 (0.2934)	0.2085 (0.3007)
R-free	0.2437 (0.3207)	0.2526 (0.3552)
Number of atoms		
macromolecules	7374	3729
ions	2	2
water	29	66
rmsd		
bond lengths (Å)	0.029	0.003
bond angles (°)	0.90	0.75
Average B-factor	66.70	85.80
macromolecules	66.70	85.90
ions	73.70	86.50
water	57.90	76.10
PDB	5CO8	5CNQ

Analysis of cleavage and binding affinity with a four-way DNA junction using point mutants of CtGEN1

Selected putative active site residues of CtGEN1 were individually converted into alanine by PCR of the gene using the Q5 site-directed mutagenesis kit (BioLab) following the manufacturer's instructions. Mutations were verified by DNA sequencing. Mutant CtGEN1 was expressed and purified as a fusion with a six-histidine tag at the C-terminal end and purified by Ni-NTA column (HiTrap™) and heparin HP. Protein concentration was estimated by absorbance at 280 nm using ε^{280} = 50,600 $M^{-1}cm^{-1}$ (per monomer).

Cleavage of junction 3 radioactively [5'-^{32}P]-labeled on the x strand in 10 mM Hepes (pH 7.5), 50 mM NaCl, 5 mM $MgCl_2$, 0.1% BSA, 1 mM DTT, 10 ng/μl calf thymus DNA and 400 nM CtGEN1 at 37°C was analyzed under single-turnover conditions as described previously (Freeman et al., 2014). Substrate and products were separated by gel electrophoresis under denaturing conditions, and quantified by phosphorimaging using a Fuji BAS 1500 phosphorimager with MacBAS software. The fraction of DNA cleaved at time t (F_t) was fitted by nonlinear regression analysis to the equation:

$$F_t = F_f.(1\text{-exp}(-k_c t)) \quad \text{[eq. 1]}$$

where F_f is the fraction of DNA cleaved at the end of the reaction and k_c the rate of cleavage. Binding affinity was measured by incubation of radioactively-labeled junction 3 with increasing concentrations of CtGEN1 in 10 mM Hepes (pH 7.5), 50 mM NaCl, 1 mM $CaCl_2$, 0.1% BSA, 1 mM DTT, 10 ng/μl calf thymus DNA for 1 h. at 20°C as described previously (Freeman et al., 2014). Bound and unbound DNA were separated by gel electrophoresis under non-denaturing conditions, and quantified by phosphorimaging using a Fuji BAS 1500 phosphorimager with MacBAS software. Data were analyzed as fraction DNA bound (f_b) versus protein concentration and fitted by non linear regression analysis to the equation :

$$f_b = 1/(1+ (K_d/ P_t)^n) \quad \text{[eq. 2]}$$

where K_d is the dissociation constant, P_t is the total protein monomer concentration. Wild type and mutant CtGEN1 concentrations were 43, 21.4, 19.3, 17.1, 15, 12.8, 10.7, 8.6, 6.4, 4.3 and 2.1 nM and the total concentration of junction 3 was 180 pM.

DNA sequences of strands contained in junction 3 used to study cleavage activity and binding of CtGEN1 mutants, and chemical probing by potassium permanganate :

b-strand : GGCTAAGGGATCCGTCCTAGCAAGGGGCTGCTACCGGAGGCTTACATCGG
h-strand : CCGATGTAAGCCTCCGGTAGCAGCCTGAGCGGTGGTTGGATGTTGACTGC
r-strand : GCAGTCAACATCCAACCACCGCTCAACTCAACTGCAGTCTAGATGGACTG
x-strand : CAGTCCATCTAGACTGCAGTTGAGTCCTTGCTAGGACGGATCCCTTAGCC

Comparative gel electrophoresis

The six possible DNA junctions with the core sequence of junction 3 (Duckett et al., 1988) with two long (40 bp) and two short (14 bp) arms were generated by chemical synthesis and hybridization in appropriate combinations of four strands (Lilley, 2008). Strands were radioactively-[5'-^{32}P]-labeled using T4 polynucleotide kinase (Fermentas) and [γ-^{32}P]ATP (Perkin Elmer). After hybridization by slow cooling, the junction species were purified by electrophoresis in a 6% (29:1) polyacrylamide gel in 90 mM Tris.borate (pH 8.5), 2 mM EDTA, recovered by electroelution and precipitated with ethanol. A fraction of each junction (3 to 10 nM) was incubated with 100 nM CtGEN1 in 10 mM Hepes (pH 7.5), 50 mM NaCl, 0.1% BSA, 1 mM DTT, 5 mM $CaCl_2$ plus 10 ng/ml calf thymus DNA. The six species were then loaded onto a 5% (37:1) polyacrylamide gel in 90 mM Tris.borate (pH 8.5), 2 mM $CaCl_2$. Electrophoresis was performed for 14 h. at 120 V at room temperature, with continuous buffer recirculation at 1 l. h^{-1}. Gels were dried and subjected to phosphorimage analysis using a BAS 1500 phosphorimager with MacBAS software (Fuji).

DNA sequences used in comparative gel electrophoretic analysis :

b strand 80 nt complete :
CGAATTCGACAGGAACCTCGAGGGATCCGTCCTAGCAAGGGGCTGCTACCGGAAGCTTACAGATGTCT
TGCGGGGATCCG
b strand 54 nt 5' end : CGAATTCGACAGGAACCTCGAGGGATCCGTCCTAGCAAGGGGCTGCTACCGGAA
b strand 54 nt 3' end : CCGTCCTAGCAAGGGGCTGCTACCGGAAGCTTACAGATGTCTTGCGGGGATCCG
b strand 14 nt central section : CCGTCCTAGCAAGGGGCTGCTACCGGAA
h strand 80 nt complete :
CGGATCCCCGCAAGACATCTGTAAGCTTCCGGTAGCAGCCTGAGCGGTGGTTGAATTCACAGATGACTG
TTCTGAATTCG
h strand 54 nt 5' end : CGGATCCCCGCAAGACATCTGTAAGCTTCCGGTAGCAGCCTGAGCGGTGGTTGA
h strand 54 nt 3' end : TTCCGGTAGCAGCCTGAGCGGTGGTTGAATTCACAGATGACTGTTCTGAATTCG
h strand 14 nt central section : TTCCGGTAGCAGCCTGAGCGGTGGTTGA

r strand 80 nt complete :

CGAATTCAGAACAGTCATCTGTGAATTCAACCACCGCTCAACTCAACTGCAGTCTAGAACACATGTCCA
CAATGGATCCG

r strand 54 nt 5' end : CGAATTCAGAACAGTCATCTGTGAATTCAACCACCGCTCAACTCAACTGCAGTC

r strand 54 nt 3' end : TCAACCACCGCTCAACTCAACTGCAGTCTAGAACACATGTCCACAATGGATCCG

r strand 14 nt central section : TCAACCACCGCTCAACTCAACTGCAGTC

x strand 80 nt complete :

CGGATCCATTGTGGACATGTGTTCTAGACTGCAGTTGAGTCCTTGCTAGGACGGATCCCTCGAGGTTCC
TGTCGAATTCG

x strand 54 nt 5' end : CGGATCCATTGTGGACATGTGTTCTAGACTGCAGTTGAGTCCTTGCTAGGACGG

x strand 54 nt 3' end : GACTGCAGTTGAGTCCTTGCTAGGACGGATCCCTCGAGGTTCCTGTCGAATTCG

x strand 14 nt central section : GACTGCAGTTGAGTCCTTGCTAGGACGG

Permanganate probing of the DNA junction

Junction 3 with 25 bp arms radioactively [5'-^{32}P]-labeled on a given strand was subjected to permanganate probing. 10 nM junction with and without 70 nM CtGEN1 was reacted with 1 mM $KMnO_4$ for 2 min in 10 mM Hepes (pH 7.5), 50 mM NaCl, 1 mM $CaCl_2$, 0.1% BSA in a volume of 100 μl at 25ºC. The reaction was terminated by addition of 25 μl of 1 M β-mercaptoethanol, 1M NaCl, 10 mM EDTA, and the DNA precipitated with ethanol. The DNA was recovered by centrifugation, resuspended in 1 M piperidine and incubated at 95ºC for 30 min. After lyophilization the DNA was dissolved in 20 μl water and lyophilization repeated two times. Reactions were performed in the same conditions with the single-stranded h or x strand to generate a thymine-specific sequence marker. The products were separated by electrophoresis in a 10% polyacrylamide gel in TBE containing 8 M urea. The dried gel was exposed to a storage phosphor screen, and the radioactive DNA visualized by phosphorimaging using a Fuji BAS 1500 phosphorimager with MacBAS software.

Fluorescence spectroscopy

Junction 3 with 25 bp arms and its corresponding r-strand resolution cleavage product were prepared with adenine nucleotides substituted by 2-aminopurine at selected single positions. Steady-state fluorescence emission spectra were recorded on an SLM-Aminco 8100 fluorimeter, with excitation at 315 nm in order to minimize the fluorescence of the CtGEN1 protein, which contains seven tryptophan residues. Fluorescence emission was recorded between 330 nm and 460 nm in 1 nm intervals. The excitation and emission monochromators were each set with band passes of 8 nm. Polarization filters were set at the magic angle, and spectra were corrected for lamp fluctuations. All the measurements were performed at 10 °C in a 5 mm × 5 mm cuvettes. Samples contained 200 nM DNA substrate in 20 mM Hepes (pH 7.5), 50 mM NaCl, 1 mM $CaCl_2$, in a total volume of 400 µl. CtGEN1 aliquots were added to final concentrations of 0, 200, 400, 600, 800 nM, 1, 1.2, 1.4 μM for the junction, and 0, 100, 200, 300, 400, 600, 800 nM, 1, 1.2, 1.4 μM for the product DNA. All fluorescence emission spectra and fluorescence intensities were corrected for tryptophan fluorescence and background emission by subtraction of control spectra, using DNA species lacking 2-aminopurine substitution. Spectra were integrated between 370 and 410 nm to calculate binding curves.

2-aminopurine-containing DNA sequences used for fluorescence spectroscopy :
Junction 3 variants were assembled by annealing three unmodified oligonucleotides with a single 2-aminopurine-containing strand. Product variants were assembled by annealing the truncated h and x strands with a 2-aminopurine-containing r strand. In the following sequences, **P** denotes 2-amino-purine and p denotes a 5'-phosphate.

b strand (50 nt) : CCTCGAGGGATCCGTCCTAGCAAGGGGCTGCTACCGGAAGCTTACAGATG

h strand (50 nt) : CATCTGTAAGCTTCCGGTAGCAGCCTGAGCGGTGGTTGAATTCACAGATG

r strand (50 nt) : CATCTGTGAATTCAACCACCGCTCAACTCAACTGCAGTCTAGAACACATG

x strand (50 nt) : CATGTGTTCTAGACTGCAGTTGAGTCCTTGCTAGGACGGATCCCTCGAGG

b strand 2AP at -3 (50 nt) : CCTCGAGGGATCCGTCCTAGCA**P**GGGGCTGCTACCGGAAGCTTACAGATG

h strand 2AP at 3: (50 nt) : CATCTGTAAGCTTCCGGTAGCAGCCTG**P**GCGGTGGTTGAATTCACAGATG

r strand 2AP at -1 (50 nt) : CATCTGTGAATTCAACCACCGCTC**P**ACTCAACTGCAGTCTAGAACACATG

r strand 2AP at +1: (50 nt) : CATCTGTGAATTCAACCACCGCTCA**P**CTCAACTGCAGTCTAGAACACATG

h strand (27-50 nt product) : p-GAGCGGTGGTTGAATTCACAGATG

x strand (1-26 nt product) : CATGTGTTCTAGACTGCAGTTGAGTC

References

Adams, P.D., Afonine, P.V., Bunkoczi, G., Chen, V.B., Davis, I.W., Echols, N., Headd, J.J., Hung, L.W., Kapral, G.J., Grosse-Kunstleve, R.W., *et al*. (2010). PHENIX: a comprehensive Python-based system for macromolecular structure solution. Acta Crystallogr D Biol Crystallog *66*, 213-221.

Beaucage, S.L., and Caruthers, M.H. (1981). Deoxynucleoside phosphoramidites - a new class of key intermediates for deoxypolynucleotide synthesis. Tetrahedron Lett. *22*, 1859-1862.

DeLano, W.L. (2002). The PyMOL Molecular Graphics System (Palo Alto, CA, DeLano Scientific).

Duckett, D.R., Murchie, A.I.H., Diekmann, S., von Kitzing, E., Kemper, B., and Lilley, D.M.J. (1988). The structure of the Holliday junction and its resolution. Cell *55*, 79-89.

Emsley, P., Lohkamp, B., Scott, W.G., and Cowtan, K. (2010). Features and development of Coot. Acta Cryst D *66*, 486-501.

Freeman, A.D.J., Liu, Y., Déclais, A.-C., Gartner, A., and Lilley, D.M.J. (2014). GEN1 from a thermophilic fungus is functionally closely similar to non-eukaryotic junction-resolving enzymes J Molec Biol *426*, 3946-3959.

Lilley, D.M.J. (2008). Analysis of branched nucleic acid structure using comparative gel electrophoresis. Quart Rev Biophys *41*, 1-39.

McCoy, A.J., Grosse-Kunstleve, R.W., Adams, P.D., Winn, M.D., Storoni, L.C., and Read, R.J. (2007). Phaser crystallographic software. Journal of applied crystallography *40*, 658-674.

Sinha, N.D., Biernat, J., McManus, J., and Koster, H. (1984). Polymer support oligonucleotide synthesis XVIII: Use of β-cyanoethyl-N,N-dialkylamino/N-morpholino phosphoramidite of deoxynucleosides for the synthesis of DNA fragments simplifying deprotection and isolation of the final product. Nucleic Acids Res *12*, 4539-4557.

Murshudov, G.N., Skubak, P., Lebedev, A.A., Pannu, N.S., Steiner, R.A., Nicholls, R.A., Winn, M.D., Long, F., and Vagin, A.A. (2011). REFMAC5 for the refinement of macromolecular crystal structures. Acta Cryst D *67*, 355-367.
