# Supplementary figures and images for: Crystal Structure of a Eukaryotic GEN1 Resolving Enzyme Bound to DNA
Source: Cell Rep. 2015 Dec 10;13(11):2565–75. doi: 10.1016/j.celrep.2015.11.042 (PMC4695337; doi:10.1016/j.celrep.2015.11.042)

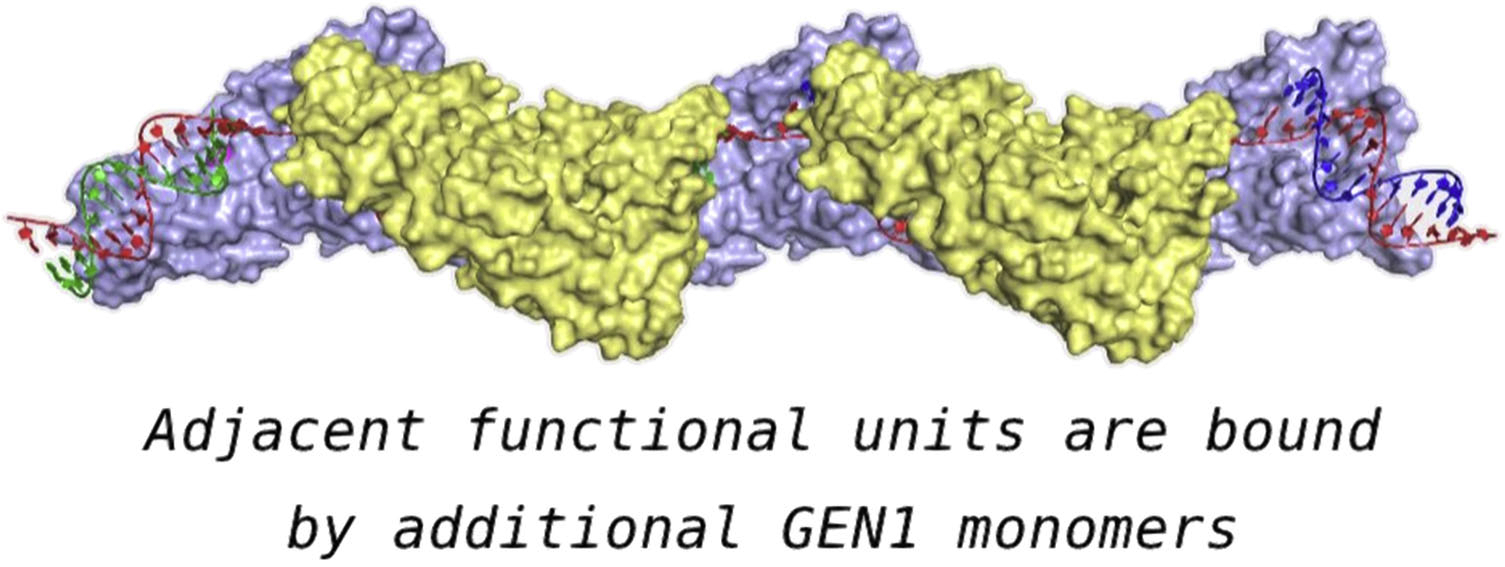

Supplement: Movie S1. The Relationship between the Asymmetric Unit, the Functional Unit, and the Lattice, Related to Figure 1 — Scene 1. The single functional unit, corresponding to the product of GEN1 cleavage. A single GEN1 monomer (blue) binds two helices connected by the continuity of the red strand. The strands are colored as in Figure 1. Scene 2. Two more functional units are added, directly repeated head-to-tail, as found in the crystal lattice. Scene 3. Two additional GEN1 monomers (yellow) bind to connect the functional units. These then rotate to show how they are bound on opposite faces of the DNA helices. Scene 4. The blue GEN1 monomers are hidden to show how the additional yellow monomers are bound in an equivalent manner to the first blue monomers. Within these complexes the two DNA helices are not connected by a central phosphate on the red strand. [file mmc2.jpg]

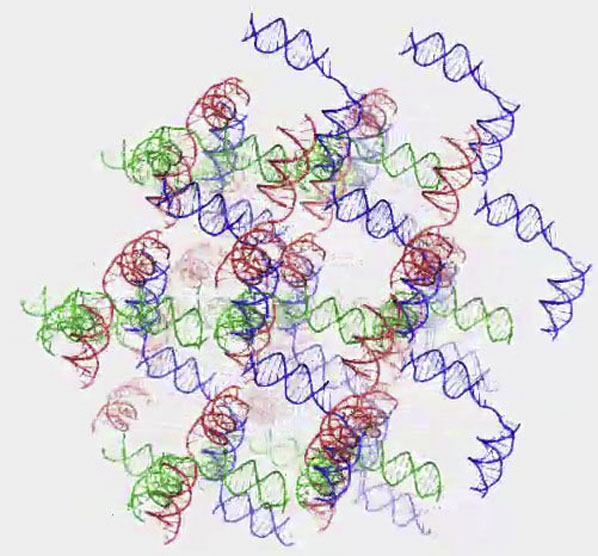

Supplement: Movie S2. The Presence of a Dimeric GEN1 Complex within the Crystal Lattice, Related to Figure 5 — Scene 1. DNA molecules in the crystal lattice, beginning by viewing down the 3-fold axis. The three linear chains of products (like that shown in Movie S1) are colored red, blue, and green, and form parallel chains through the lattice. The lattice is then rotated sequentially about orthogonal axes. Scene 2. Most of the molecules become grey, with just two product molecules left colored red and green. The view now zooms into these, and the remaining molecules fade out. Scene 3. The two DNA products juxtaposed in the lattice are rotated, and recolored to match the coloring of the strands of the product used in Figure 1A. Scene 4. Two bound CtGEN1 molecules now become visible, forming a dimeric complex interacting via a protein-protein interface. [file mmc3.jpg]

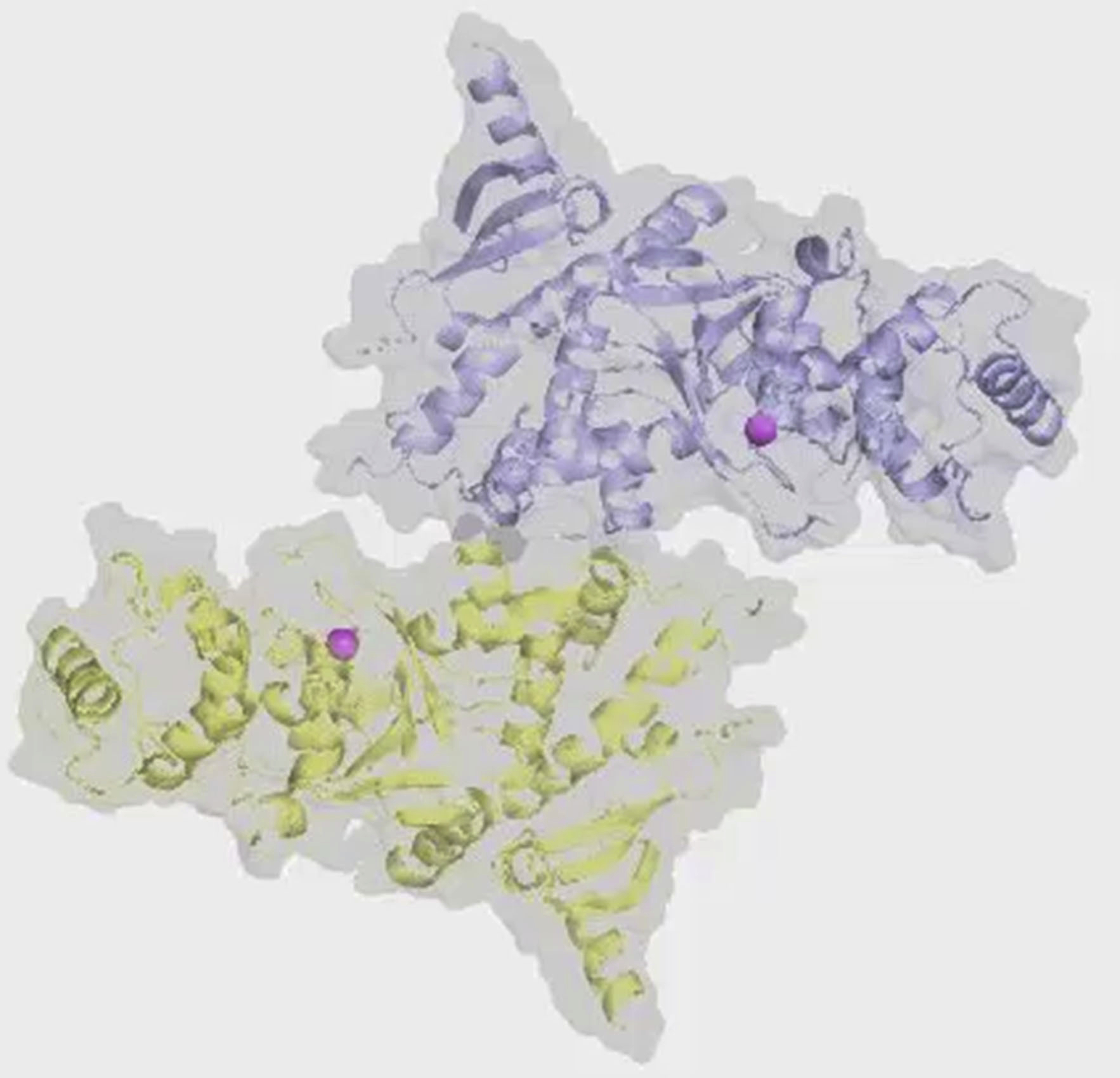

Supplement: Movie S3. The Dimer Complex, Closely Related to a Four-Way Junction Bound to Two GEN1 Monomers, Related to Figure 5 — Scene 1. The dimeric complex of CtGEN1 is shown in cartoon form. This rotates and switches to space-filling representation. Scene 2. The two bound DNA products now appear. Rotation occurs—note the view down the axis of the coaxial (uncleaved) arms as it passes, also showing that the remaining (cleaved) arms are mutually perpendicular. Scene 3. One of the two products is recolored so that the four strands are now colored as in the complete junction (Figure 1A). Scene 4. The strands become reconnected to form an intact junction. The view now zooms into the center of the junction, which is now shown with bonds in stick form. Notice that the reconnected green h strand is now close to the active site, indicated by the magenta metal ion. The strand reconnection was modeled manually—see text for details. [file mmc4.jpg]
