# Supplementary material for: Crystal Structure of a Eukaryotic GEN1 Resolving Enzyme Bound to DNA
Source: Cell Rep. 2015 Dec 10;13(11):2565–75. doi: 10.1016/j.celrep.2015.11.042 (PMC4695337; doi:10.1016/j.celrep.2015.11.042)
Supplement: Document S2. Article plus Supplemental Information [file mmc5.pdf]

Cell Reports

Article

Crystal Structure of a Eukaryotic GEN1 Resolving Enzyme Bound to DNA

Graphical Abstract

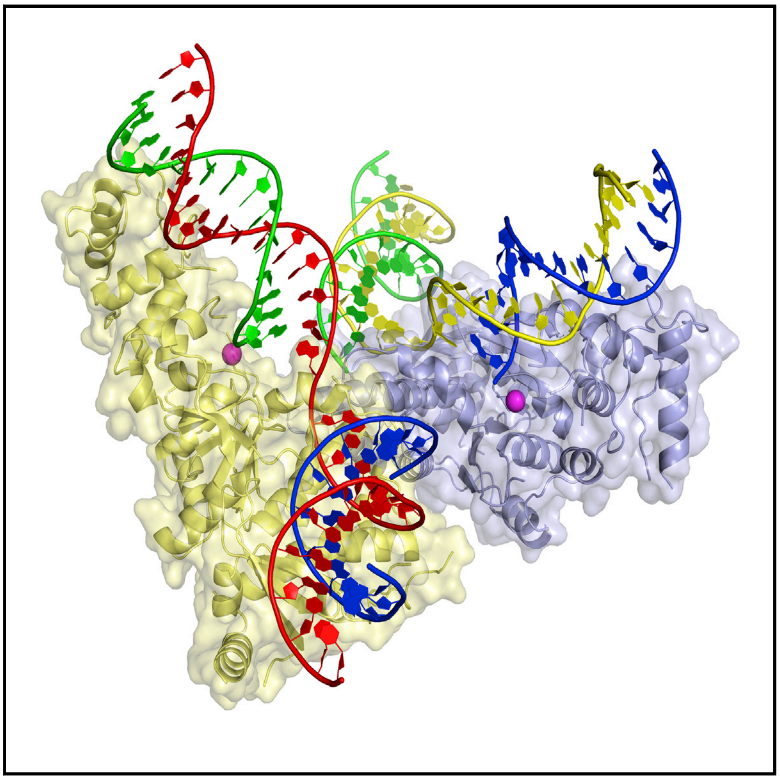

Authors

Yijin Liu, Alasdair D.J. Freeman, Anne-Cécile Déclais, Timothy J. Wilson, Anton Gartner, David M.J. Lilley

Correspondence

d.m.j.lilley@dundee.ac.uk

In Brief

Liu et al. present the crystal structure of a fungal GEN1 Holliday junction-resolving enzyme. GEN1 is bound to a product of cleavage, comprising two connected arms of the junction. Two GEN1 molecules dimerize to juxtapose two products such that they can be simply reconnected to form a junction.

Highlights

- GEN1 crystallized with a resolution product containing two perpendicular DNA helices
- GEN1 shares the FEN1 superfamily fold, with a two-metal ion-containing active site
- GEN1 forms a dimer that juxtaposes two products in a substrate-like complex
- A resulting model of a GEN1-junction complex is supported by solution experiments

Accession Numbers

5CO8
5CNQ

Liu et al., 2015, Cell Reports *13*, 2565–2575
December 22, 2015 © 2016 The Authors.
http://dx.doi.org/10.1016/j.celrep.2015.11.042

OPEN ACCESS
CellPress

Crystal Structure of a Eukaryotic GEN1 Resolving Enzyme Bound to DNA

Yijin Liu,[1] Alasdair D.J. Freeman,[1] Anne-Cécile Déclais,[1] Timothy J. Wilson,[1] Anton Gartner,[2] and David M.J. Lilley[1,*]
[1]Cancer Research UK Nucleic Acid Structure Research Group
[2]Center for Gene Regulation and Expression
MSI/WTB Complex, University of Dundee, Dow Street, Dundee DD1 5EH, UK
*Correspondence: d.m.j.lilley@dundee.ac.uk
http://dx.doi.org/10.1016/j.celrep.2015.11.042
This is an open access article under the CC BY license (http://creativecommons.org/licenses/by/4.0/).

SUMMARY

We present the crystal structure of the junction-resolving enzyme GEN1 bound to DNA at 2.5 Å resolution. The structure of the GEN1 protein reveals it to have an elaborated FEN-XPG family fold that is modified for its role in four-way junction resolution. The functional unit in the crystal is a monomer of active GEN1 bound to the product of resolution cleavage, with an extensive DNA binding interface for both helical arms. Within the crystal lattice, a GEN1 dimer interface juxtaposes two products, whereby they can be reconnected into a four-way junction, the structure of which agrees with that determined in solution. The reconnection requires some opening of the DNA structure at the center, in agreement with permanganate probing and 2-aminopurine fluorescence. The structure shows that a relaxation of the DNA structure accompanies cleavage, suggesting how second-strand cleavage is accelerated to ensure productive resolution of the junction.

INTRODUCTION

Homologous recombination plays a number of key roles in the cell. In meiosis, it creates a transient physical linkage between homologous chromosomes to ensure accurate segregation and to facilitate genetic diversity in the process. In mitotic cells, recombination provides a mechanism for the repair of DNA double-strand breaks and facilitates the repair of interstrand crosslinks and DNA lesions arising during replication. Defective homologous recombination results in significantly increased susceptibility to cancer in humans.

The central intermediate species of recombination is the four-way (Holliday) junction (Holliday, 1964), in which four DNA helices are temporarily connected by strand continuity. Processing of such junctions is a key event that can occur by dissolution or resolution. Dissolution involves translocation of two junctions toward each other by BLM helicase followed by decatenation mediated by topoisomerase IIIα (Cejka et al., 2010; Ellis et al., 1995; Wu and Hickson, 2003). By contrast, resolution involves the action of nucleases that are targeted to the structure of the four-way junction. A number of junction-resolving enzymes from bacteria and their phages, archaea, and yeast mitochondria have been well characterized (reviewed in Déclais and Lilley, 2008). These enzymes selectively bind four-way DNA junctions in dimeric form with high affinity, recognizing and manipulating the junction structure, and introducing symmetrical, bilateral cleavages that result in productive resolution.

Two main junction-resolution activities have been identified in eukaryotic cells, both unrelated to those from lower organisms. The first was GEN1, identified by West and coworkers after a long search (Elborough and West, 1990; Constantinou et al., 2001, 2002) and isolated through extensive biochemical fractionation of HeLa cells (Ip et al., 2008; Rass et al., 2010). GEN1 was also isolated from budding yeast (Ip et al., 2008) (as Yen1) and *Caenorhabditis elegans* (Bailly et al., 2010). The other main activity arises from the combination of SLX1-MUS81-EME1-SLX4 proteins (Agostinho et al., 2013; Andersen et al., 2009; Fekairi et al., 2009; Muñoz et al., 2009; Svendsen et al., 2009). At least one of these activities must be functional to maintain cell viability, as GEN1 and SLX4 are synthetically lethal in human cells due to dysfunctional mitosis resulting from unprocessed junctions (Garner et al., 2013). The meiotic phenotype of *mus81Δ* fission yeast is restored by ectopic expression of human GEN1 (Lorenz et al., 2010), and the budding yeast ortholog Yen1 is required to resolve persistent DNA junctions during meiosis when *mus81* is deleted (Matos et al., 2011).

GEN1 is a member of a superfamily of structure-selective nucleases (Grasby et al., 2012). These include the enzymes FEN1 that acts on various flap and double-flap structures (Ceska et al., 1996; Hosfield et al., 1998; Tsutakawa et al., 2011), EXO1 that cleaves 3′-overhang structures (Orans et al., 2011) and XPG (Rad2 in yeast) that acts in nucleotide excision repair (Miętus et al., 2014). Crystallographic structures of the latter three enzymes reveal a common fold. This contains a relatively flat platform of dimension ~70 × 30 Å, based upon a central seven-strand twisted β sheet flanked on both sides by a total of ~15 α helices, reminiscent of an elaborated Rossmann fold. All three proteins bind a DNA duplex (in FEN1 this is the helix with the 5′ flap strand), with a common element (termed H2TH) that contacts the backbone of the uncleaved strand. In FEN1, a second DNA duplex (that with the 3′ flap and connected to the duplex with the 5′-flap by the continuity of the uncleaved strand) is bound, such that the two axes are virtually perpendicular. The active site is centrally located on the platform and

CrossMark

comprises a number of conserved carboxylate residues around the α-β interface that coordinate one or two metal ions in the crystal structures. Mechanistic studies on phage T5 FEN indicate that at least two metal ions participate in the hydrolysis reaction (Syson et al., 2008). Some functionally important helices project above the platform. In all cases, the α helix immediately C-terminal to the second section of β sheet (counted from the N terminus) projects above the β sheet region to abut the end of the DNA duplex, splaying apart the strands; it is thus termed the helical wedge. This helix directs a conserved tyrosine toward the DNA in FEN1 (Tsutakawa et al., 2011). A second section of polypeptide observed in FEN1 and EXO1 is directed above the platform, comprising ~40 amino acids in two α helices. This is termed the helical arch, through which passes the single strand of the substrate thereby selecting substrates with such features.

Although sequence homology indicates that GEN1 will contain a number of features in common with FEN1, EXO1, and XPG, we would anticipate that it would have to differ from the other family members in a number of key respects. Given that GEN1 resolves a four-way DNA junction, it must act in dimeric form, in common with all known junction-resolving enzymes (Déclais and Lilley, 2008), in agreement with our recent analysis (Freeman et al., 2014). Since the strands of a four-way junction are base-paired and covalently continuous, it would not require the helical arch, so this feature would probably be extensively modified in GEN1. Although the active site would be expected to be conserved in GEN1, the key question of interest is how the enzyme is selective for DNA junctions. To answer this, we require a molecular structure of the protein bound to DNA.

Studies of GEN1 from human cells have shown that the N-terminal section acts in dimeric form to resolve four-way DNA junctions (Rass et al., 2010). However, all fragments of the enzyme have been found to be poly-disperse and fail to form discrete complexes with junctions. In contrast, we found that the orthologous enzyme from the thermophilic fungus *Chaetomium thermophilum* was very well behaved (Freeman et al., 2014). In free solution the protein exists primarily in monomeric form, but binds to DNA junctions as a discrete dimer to generate bilateral cleavage by accelerating second strand cleavage. The biochemical properties of this enzyme conform closely to those established for the junction-resolving enzymes as a class (Déclais and Lilley, 2008). We have now solved a crystal structure of active GEN1 from *C. thermophilum* bound to the DNA resulting as the product of cleavage.

RESULTS

Crystallization and Structure Determination of CtGEN1

The N-terminal 1–487 wild-type amino acid sequence of *C. thermophilum* GEN1 protein (hereafter referred to as CtGEN1) with a C-terminal six-histidine tag was expressed in *Escherichia coli*, using normal and selenomethionine-containing medium.

Purified CtGEN1 was mixed in equimolar quantities with a four-way DNA junction based on the well-characterized junction 3 (Duckett et al., 1988) and comprising 15 bp in each helical arm. Equal volumes of DNA and protein were mixed in a final concentration of 100 mM HEPES (pH 7.5), 2 mM $MgCl_2$, 20% PEG10000, and incubated with the same buffer using hanging drop vapor diffusion at 7°C. Experimental phasing was achieved by single-wavelength anomalous dispersion using the selenomethionine-substituted CtGEN1 (PDB: 5CO8). Some crystals were soaked in 1 mM $MnCl_2$ solution to exchange Mg^{2+} with Mn^{2+} ions (PDB: 5CNQ). The two crystal forms were solved at a resolution of 2.5 and 2.6 Å, respectively. Crystallographic statistics are presented in the Supplemental Information.

Analysis of the DNA Present in the Crystals

The presence of $MgCl_2$ in the crystallization solution induced cleavage of the four-way DNA junction by CtGEN1. In principle, resolution cleavage could lead to four possible products, resulting from cleavage of either pair of two opposing strands (i.e., either strands b and r or strands h and x, Figure 1A). However, our previous biochemical experiments show there is a very strong bias toward cleavage on the h and x strands of junction 3 (Freeman et al., 2014), giving just two products (in which the b and r strands remain intact). Electrophoretic analysis of the DNA contents of our crystals (Figure S1A) shows that the crystallization has selected one of the two products, i.e., that with the intact r strand. The junction used in crystallization trials was assembled from four 30 nt strands that formed four 15 bp helices and had an asymmetric core sequence that precluded branch migration. Given that CtGEN1 cleaves strands 1 nt 3′ of the junction, the crystallized product should comprise a 14 bp helix (the 3′ 14 nt of the h strand and the 5′ 14 nt of the r strand) and a 15 bp helix (the 3′ 15 nt of the r strand and the 5′ 15 nt of the x strand), with a mismatch between the two helices comprising nt 15 of the r strand and nt 16 of strand x (Figure 1A).

The CtGEN1-DNA Complex in the Crystal Lattice

The crystals belong to the $P3_121$ space group, in which the asymmetric unit contains one CtGEN1 monomer and a duplex of DNA with 14 and 15 nt strands (14 bp with a 3′ overhang) that adopts a standard B-form helix with no distortion. The 5′ end of the 14 nt strand is located in the active site of the enzyme, indicated by the presence of a bound Mg^{2+} ion (Figure 1B). How does this asymmetric unit arise from the crystallized product DNA that includes a 30 nt strand? The explanation must be that the two duplexes of the product occupy crystallographically equivalent positions within the lattice. The structure of the asymmetric unit arises naturally from resolution cleavage of the h strand, which produces a 14 bp helix with the 5′ end of the 14 nt h strand in the CtGEN1 active site and a mismatched 3′ nucleotide on the r strand. Within the crystal lattice, the other half of the product molecule is in a crystallographically equivalent position with respect to another CtGEN1 monomer, with the 5′ end of its x strand located in the active site of the second monomer (Figure 1C; Movie S1). As a result, contributions from both halves of the product DNA become averaged in the electron density map and have been modeled with 50% occupancy (Figure S1B). The second DNA helix should comprise a 15 bp duplex with a 1 nt 3′ overhang. However, its end is frayed by insertion into the active site of the second CtGEN1 monomer and the 5′ x nucleotide is not seen. Neither is the overhanging 3′ nt of the x strand, thus only 14 nt of the x strand are observed, making it equivalent to the h strand in the structure.

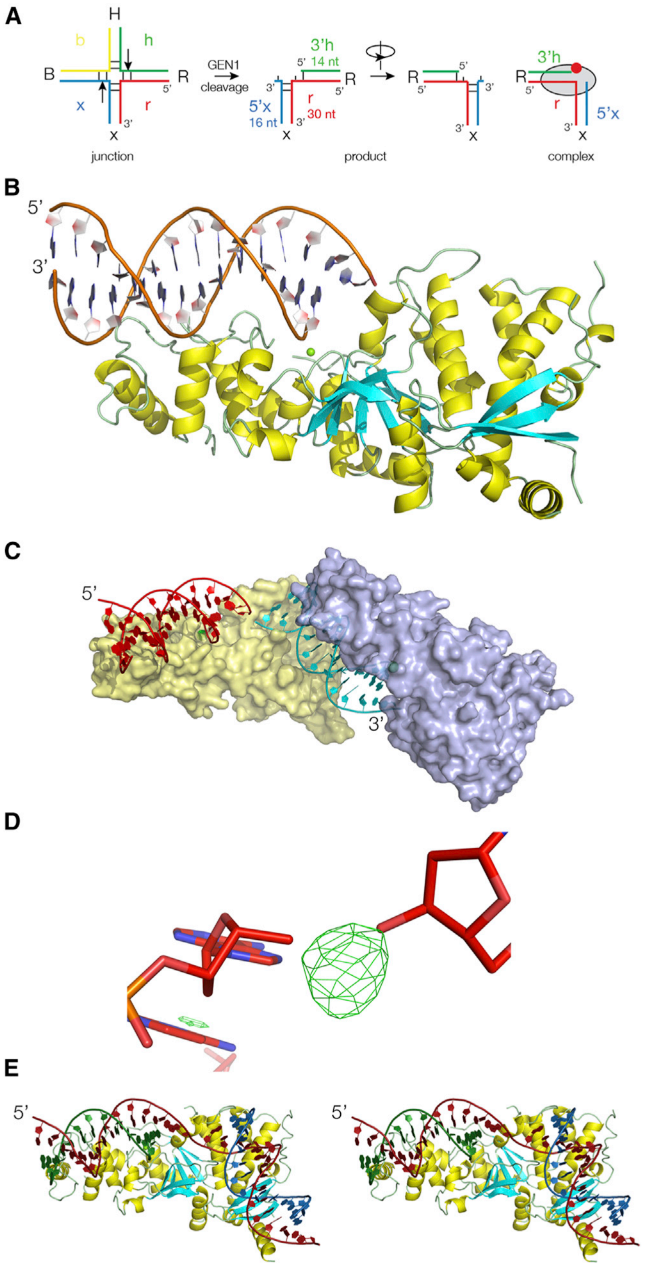

Figure 1. The Asymmetric Unit and Formation of the Functional Unit in the Lattice

(A) Scheme showing the pattern of cleavage of a four-way DNA junction by CtGEN1 and the resulting product formation. Conventionally, we name the four arms B, H, R, and X, and the component strands b, h, r, and x. The arrows show the preferred position of cleavage by CtGEN1, to generate the product (Freeman et al., 2014). This is also shown rotated by 180°to match the view seen in most of the molecular graphics. In the complex CtGEN1 is depicted as a black ellipse, with the active site indicated by the red spot. See also Figure S1B.

(B) Molecular graphics image of the asymmetric unit in the crystal lattice. This comprises a monomer of GEN1 bound to a 14 bp duplex of DNA with a 1 nt 3′ overhang.

(C) Two asymmetric units, showing how each DNA is bound by two GEN1 monomers, colored yellow and blue. See also Movie S1.

(D) F_o-F_c simulated annealing omit map of the electron density at the interface of the two duplexes. This was calculated by omission of the central phosphate of the long strand in the model used to refine the structure. Density corresponding to a phosphate group with partial occupancy is present, clearly linking the two strands. This is consistent with the existence of a 30 nt strand in the crystal. See also Figure S1A.

(E) Parallel-eye stereo molecular graphics image of the functional unit comprising one GEN1 monomer and a product that includes a 30 nt DNA strand. The strands are colored to be consistent with the scheme in (A) with the r strand as the 30 nt strand.

The axes of the two duplexes forming a product molecule bound to one CtGEN1 subunit are virtually perpendicular. In the crystal lattice, a second monomer brings together the distal ends of duplexes from two adjacent products at a 90° angle (yellow monomer in Figure S1B and Movie S1). The 5′ end of the r strand of one product is juxtaposed with the 3′ end of the r strand of an adjacent product such that the r strand appears quasi-continuous between the two product molecules. Inspection of an $\mathbf{F_o}$-$\mathbf{F_c}$ electron density map calculated from a model lacking the central phosphate reveals clear density corresponding to a phosphate connecting the two halves of the 30 nt long r-strand with partial occupancy (Figure 1D), in agreement with the expected averaging between central and distal ends of the duplexes. The head-to-tail GEN1-bound products form parallel chains running through the crystal lattice in three orientations related by the trigonal symmetry (Movie S2). However, these parallel chains are not all in register; the products in one strand could align with those of an adjacent strand, or they could be offset such that the h strands in one chain align with the x strands of an adjacent strand. This is because all of the contacts within the lattice are protein-mediated, and GEN1 binds each half of the product in an equivalent manner. This lack of alignment results in the averaging of the DNA sequences in the electron density map.

The Functional Unit Is a CtGEN1-Product Complex

The functional unit is thus one CtGEN1 monomer with one complete DNA product. We have, therefore, associated the 30 nt strand as one covalently continuous strand within the complex and will represent it in that manner hereafter. Thus the functional unit contains a product of resolution (Figure 1E), comprising a 30 nt r strand, the 14 nt 3′ section of the h strand (referred to subsequently as 3′h) and the 16 nt 5′ section of the x strand (5′x) of which the first and last nucleotides are not visible in the electron density. The trajectory of the DNA is strikingly similar to that bound to human FEN1 (hFEN1) (Tsutakawa et al., 2011). The DNA from the two complexes can be superimposed with a root-mean-square deviation (RMSD) = 2.88 Å (Figure 2D).

The Structure of the CtGEN1 Monomer

We have fitted 84% of the amino acids in the calculated electron density maps, modeling all the secondary structure except for a number of disordered loops and the C-terminal 22 amino acids. CtGEN1 is an approximate hemi-ellipsoid of dimensions 80 × 30 × 30 Å, broadening at one end to 40 Å (Figure 2A). The shape can be likened to a rowing boat, with the DNA bound on one edge of the relatively flat surface. The connectivity of the

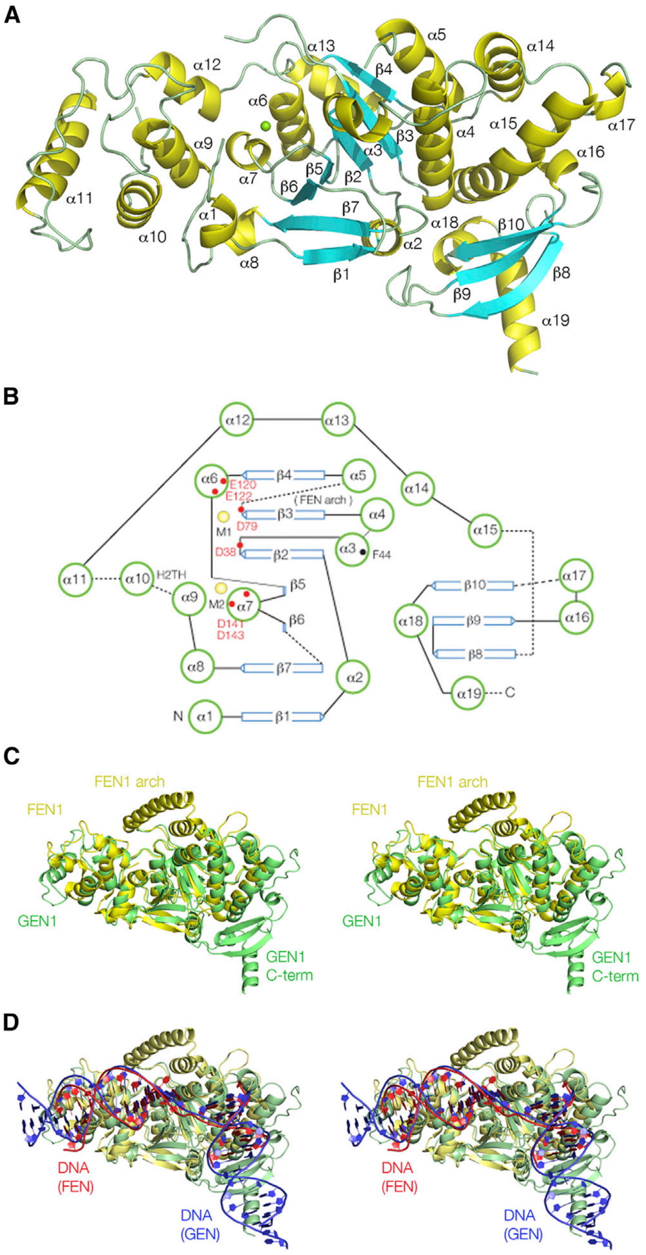

Figure 2. The Structure of the CtGEN1 Protein

(A) Molecular graphics image of CtGEN1 with the secondary structure indicated by yellow (α helix), cyan (β sheet), and green (coil regions). The green sphere is the Mg^{2+} ion bound in the active site of the enzyme.

(B) Scheme showing the connectivity of the secondary structure and the location of key residues. Two bound metal ions are shown as yellow spheres. Broken lines indicate short connecting segments that are not visible in the electron density map—these have been left blank in (A). The section connecting β3 and α5 comprising 12 amino acids corresponds to the helical arch region of FEN1.

(C) Parallel-eye stereoscopic view of superposed structures of CtGEN1 and human FEN1 (PDB: 3Q8L) (Tsutakawa et al., 2011). GEN1 is shown in green and FEN1 in yellow.

(D) Parallel-eye stereoscopic view of superposed structures of CtGEN1 and hFEN1 with their bound DNA. The DNA of CtGEN1 is blue and that of FEN1 is red. See also Figure S2.

secondary structure is shown diagrammatically in Figures 2B and S2. This can be considered in two sections. The N-terminal section runs from α1 to α15. It is constructed around a central seven-strand β sheet that is parallel except for β7. It is flanked on both sides by α helices, including the four-helix bundle comprising α4 and α5 together with α14 and α15 that buries an area of 1,256 $Å^2$. The connectivity of helices and sheet is in most respects identical to that of the FEN1 family members. The most prominent difference between CtGEN1 and hFEN1 is that the two helices forming the helical arch in the latter are not observed in CtGEN1. These helices would lie between β3 and α5 in CtGEN1; if they adopted a regular structure they would be visible, yet that section cannot be observed in the electron density map consistent with it being unstructured and flexible. Moreover, there are only 12 amino acids located between β3 and α5 in CtGEN1 whereas the helical arch of hFEN1 comprises 39 amino acids. If this section is excluded from hFEN1, then it and the observed sections of CtGEN1 from the N terminus to α15 superimpose with an RMSD = 2.07 Å (Figure 2C), showing that the two proteins are closely related in structure.

The C-terminal section of CtGEN1 contains a three-strand antiparallel β sheet (β8–10) and four α helices. This section fills the wider end of the structure (the stern in the rowing boat analogy) and has no counterpart in the other FEN1-XPG family members. Submission to the Dali server (Holm and Rosenström, 2010) indicates that the structure is similar to a series of chromobox homology proteins. The two sections of CtGEN1 are connected by a disordered 15 amino acid peptide not observed in the electron density. The C terminus of CtGEN1 lies at the end of α19, directed away from the protein. In the full-length GEN1 this would connect to the remaining section of protein, of largely unknown function. We find that we can fuse additional sections of proteins C-terminal to residue 530 without loss of enzyme activity. By contrast, the N terminus of CtGEN1 is located at a DNA-protein interface and only 4 Å from the active site. Any modification of the N terminus results in complete loss of activity (data not shown).

DNA-Protein Contacts in the Complex

Within the functional unit, each CtGEN1 monomer is bound to one product DNA molecule, derived from two arms of the four-way junction as the product of resolution cleavage (Figure 1A). The DNA-binding face of CtGEN1 contains a number of lysine and arginine residues, forming a predominantly electropositive (i.e., basic) track especially at the points of backbone contact (Figure 3A), burying a surface area of 1,394 $Å^2$. There are no sequence-specific contacts observed with the DNA nucleobases. Altogether, both DNA helices are held by multiple points of attachment to both strands (Figure S3), constraining their relative perpendicular orientation in the complex.

The cleaved DNA helix (the R arm of the product, i.e., the helix containing the 5′ end of the 30 nt r strand) is bound at the "bow" end of the CtGEN1 molecule. A complete turn of DNA is bound along one edge of the flat platform generated by helices α1, α9, α10, and α12 and their connecting loop regions. Helices α9 plus α10 correspond to the H2TH motif observed in hFEN1 (Tsutakawa et al., 2011). In addition, helix α10 of the H2TH motif is oriented directly at the backbone, with its N-terminal end 4 Å

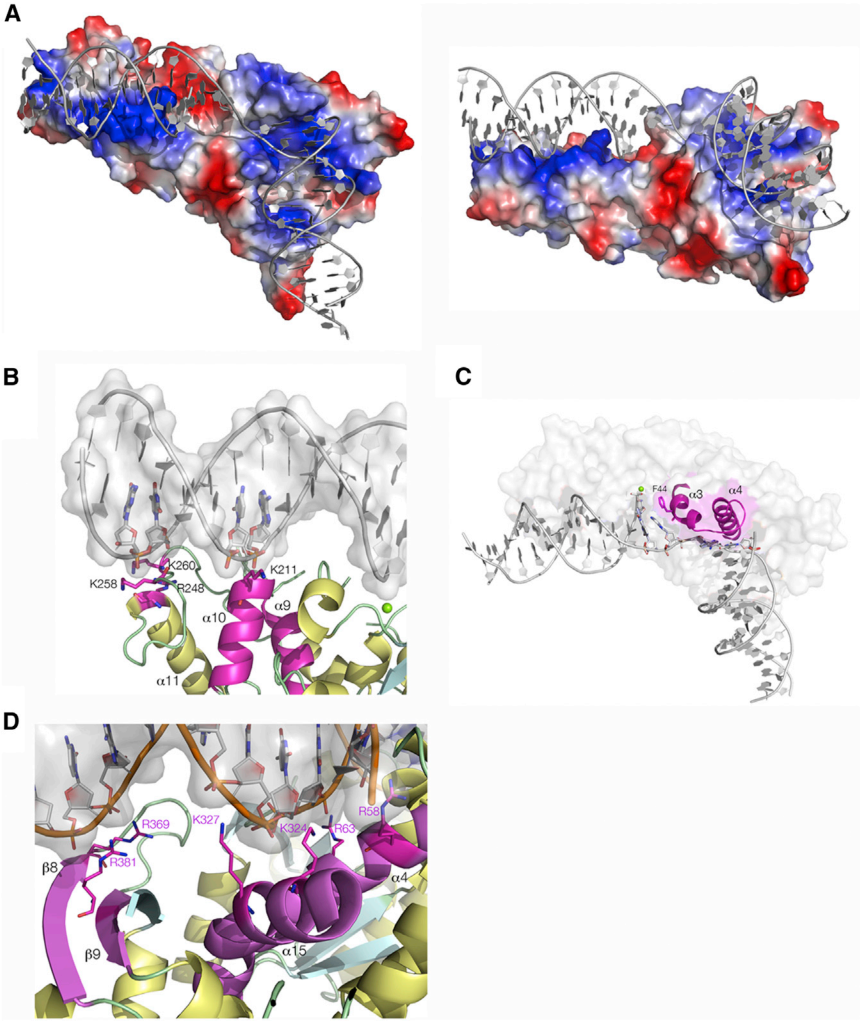

Figure 3. DNA-Protein Interactions in the CtGEN1-Product Complex

(A) Molecular graphics images of the functional unit of the GEN1-product complex with the electrostatic surface of the protein shown. Electropositive areas are shown blue and electronegative areas shown red. Two different views are shown.

(B) DNA-protein contacts in the cleaved arm of the product including the H2TH element (α9 and α10, highlighted magenta). A number of basic residues contact the phosphate groups on inward-facing strands of DNA.

(C) The central region of the complex, where the helical wedge (α3 and α4) abuts the ends of the DNA helices at the junction. The phenyl side chain of F44 is stacked with the unpaired base on the continuous strand.

(D) DNA-protein contacts in the uncleaved arm of the product. α4, α15, β8, and β9 (highlighted magenta) contribute basic residues that contact phosphate groups on both strands on the protein-facing side of the DNA.

A schematic of all the protein-DNA contacts is shown in Figure S3.

from a phosphate group of the non-cleaved strand such that its charge is partially neutralized by the positive pole of the helix dipole (Figure 3B).

Helix α3 is positioned at the end of the cleaved helix, incompatible with the continuation of double-stranded helical geometry, and helix α4 is similarly positioned at the end of the uncleaved helix (Figure 3C). The unit comprising α3, α4, and the connecting coil region is functionally equivalent to the helical wedge of hFEN1. The phenyl side chain of F44 on α3 is stacked with the unpaired nucleobase at the hinge of the unbroken r strand; this position aligns with Y40 in hFEN1, where it has a similar function. The strand connecting the two duplexes passes between the helical wedge α3 and α4 and the loop between β6 and β7.

The uncleaved duplex (the X arm of the product, containing the 3′ end of the unbroken r strand) is located in a strongly electropositive cleft, with backbone contacts between the r strand and α4 and α15 and the 5′x strand with the three-strand β sheet (Figure 3D). These contact 8 bp in total, on one face of the DNA helix. The C-terminal section of CtGEN1 not found in hFEN1 contacts an additional half-turn of DNA, thus adding substantially to the contacts on that arm.

The Active Site

The 5′ end of the 3′ h strand (i.e., the site of nucleolytic cleavage by the resolving enzyme) is directed down into a strongly electronegative cavity near the center of the enzyme that we assign to be the active site of the enzyme (Figure 4). The cavity contains six conserved acidic amino acids, contributed by the N-terminal ends of α6, α7, and the C-terminal ends of β2 and β3. A seventh conserved acidic residue (D199) is located in a disordered loop. Metal ions are bound in the middle of this cluster of acidic side chains. For the original Mg^{2+} crystals, a single bound ion (M2) was observed (Figure 4A), but after soaking the crystals with $MnCl_2$, two bound metal ions were observed (Figures 4B and S4). Metal ion M2 is 2.1 Å from the carboxylate groups of D141 and D143, while M1 is 2.2 Å from the carboxylate group of E122, 4.1 Å from that of D38 and D79, and 4.7 Å from E120. Substitution of each acidic residue individually to alanine (Table S1) shows that while DNA binding is almost unaffected by removal of the carboxylate groups, cleavage activity is impaired for each mutant, in most cases by orders of magnitude. However, the impairment of activity for the D38A mutant is relatively small. It is very likely that the metal ions adjust their position within the pocket during the course of binding of the four-way junction, as the phosphodiester group becomes directly coordinated and the reaction progresses through the cleavage of the two strands. In the standard two-metal ion model of phosphoryl transfer reactions (Steitz and Steitz, 1993), the metal ions serve to activate the water nucleophile, stabilize the anionic transition state and position the reactants. Nucleases operating such a mechanism frequently use a positively charged side chain to stabilize the transition state,

OPEN
ACCESS
CellPress

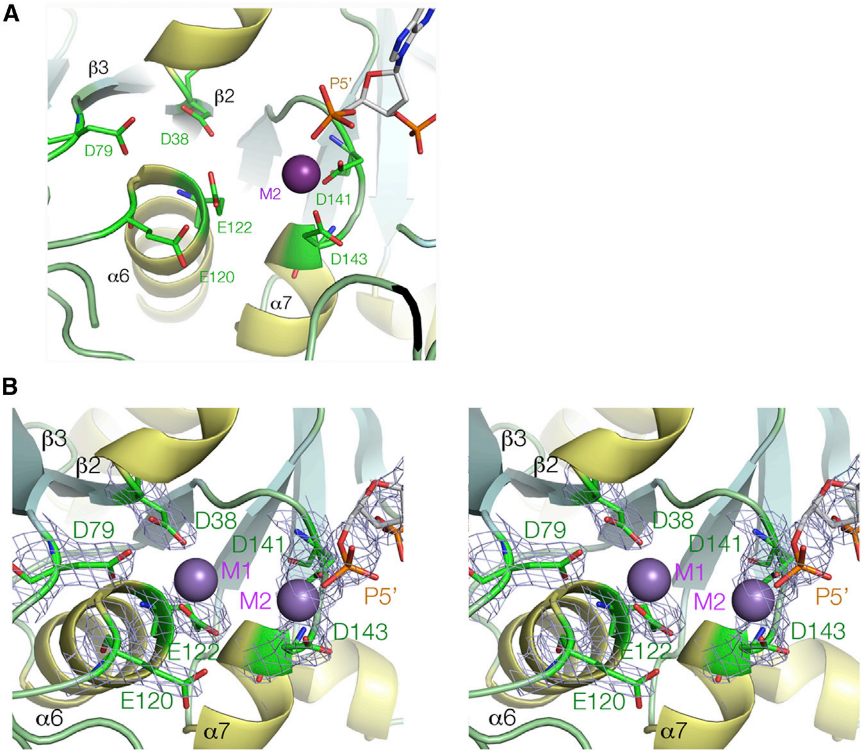

Figure 4. Molecular Graphics Images of the Active Site of CtGEN1

The active site comprises six carboxylate side chains contributed by β2, β3, α6, and α7 that coordinate divalent cations.

(A) The active site of CtGEN1 from a crystal grown in Mg^{2+} ions, showing a single bound metal ion.

(B) After exposure of the crystal to Mn^{2+} ions, two bound metal ions are observed. These are coordinated to the six carboxylate side chains and the terminal phosphate group (P5′). Parallel-eye stereoscopic view of the active site showing the six aspartate and glutamate amino acids and the 5′ phosphate, with the $2F_o$-F_c electron density map for these components contoured at 2σ. An anomalous scattering map for Mn^{2+} is shown in Figure S4.

exemplified by the active site lysine of the junction-resolving enzyme T7 endonuclease I (Déclais et al., 2001). There are a number of candidates for this role within the unobserved region between β3 and α5.

Interaction between CtGEN1 Monomers in the Crystal Lattice Reveals a Potential Conformation of a Dimeric Form of the Enzyme Bound to a Four-Way Junction

Examination of the crystal lattice reveals that protein-protein interaction between CtGEN1 monomers brings two bound DNA products into close proximity as though forming a four-way junction (Figures 5A, 5B, S5A, and S5B; Movie S2). The two CtGEN1 molecules interact primarily by the ends of alpha helices α4, α5, and α14 (Figures 5C, 5D, and S5C) and their associated loops, burying a surface area of 530 $Å^2$. The interaction generates an almost coaxial alignment of the uncleaved DNA helices, while the cleaved helical arms rotate toward each other on the major groove side such that they include an angle of close to 90°. An axis of 2-fold symmetry bisects the plane defined by the axes of these two helices and passes through the center of, and is normal to, the coaxially aligned helices. Within this complex, it is possible to reconnect the 5′ ends of the h strands with the 3′ ends of the x strands to generate a covalently intact four-way junction (Figure 5E; Movie S3). This requires the base pair located at the junction-proximal end of the uncleaved helix to be broken and unstacked, the nucleotide at the 3′ end of the x strand to be rotated around toward the h strand and the (unobserved) 16th nucleotide of the x strand to be modeled to make the phosphodiester linkage (Figure S6). These two nucleotides lie close to and may stabilize the disordered amino acids between β3 and α5.

The Shape of a DNA Junction Bound to CtGEN1 Observed in Solution

We have investigated the overall shape of the complex of CtGEN1 bound to an intact four-way DNA junction in solution using comparative gel electrophoresis (Lilley, 2008). This method was originally used to determine the structure of the four-way DNA junction in free solution (Duckett et al., 1988) and has been extended to study the shape of junctions bound to junction-resolving enzymes (Duckett et al., 1995; Giraud-Panis and Lilley, 1998; Pöhler et al., 1996; White and Lilley, 1996, 1997). In this method, we compare the electrophoretic mobility of the six possible forms of a junction with two long (here 40 bp) and two short (14 bp) arms. The global shape of the junction can be deduced from the symmetry and pattern of the relative mobilities of the different species, since the relative mobility of species increases with the angle included between the long arms.

Six long-short arm species of junction 3 comprising all combinations of two long and two short arms were constructed, each from four synthetic radioactively [5′-^{32}P]-labeled DNA strands. The six species are named according to the long arms. A fraction of each junction was incubated with a molar excess of CtGEN1 to form an enzyme-junction complex. These were then loaded on to a 5% polyacrylamide gel and electrophoresis was performed under non-denaturing conditions in the presence of 50 mM NaCl and 2 mM $CaCl_2$; these conditions induce folding of the junction but inactivate the nuclease activity of the enzyme.

The junctions in complex with CtGEN1 migrate significantly more slowly than free junctions and exhibit a completely different pattern of relative mobility compared to a free junction (Duckett et al., 1988). The pattern of the complexes comprises five species migrating at an equal, slower rate, with just one species (HX) of significantly faster mobility (Figure 6). Species RX corresponds to that seen in the crystal, and thus the long R and X helices should include 90°. Species HR has virtually identical mobility so should also include 90°. In Euclidian geometry, if RX = HR = 90° this requires species HX (i.e., the fast-migrating species) to include 180°. Species BH and BX also have closely

OPEN ACCESS
CellPress

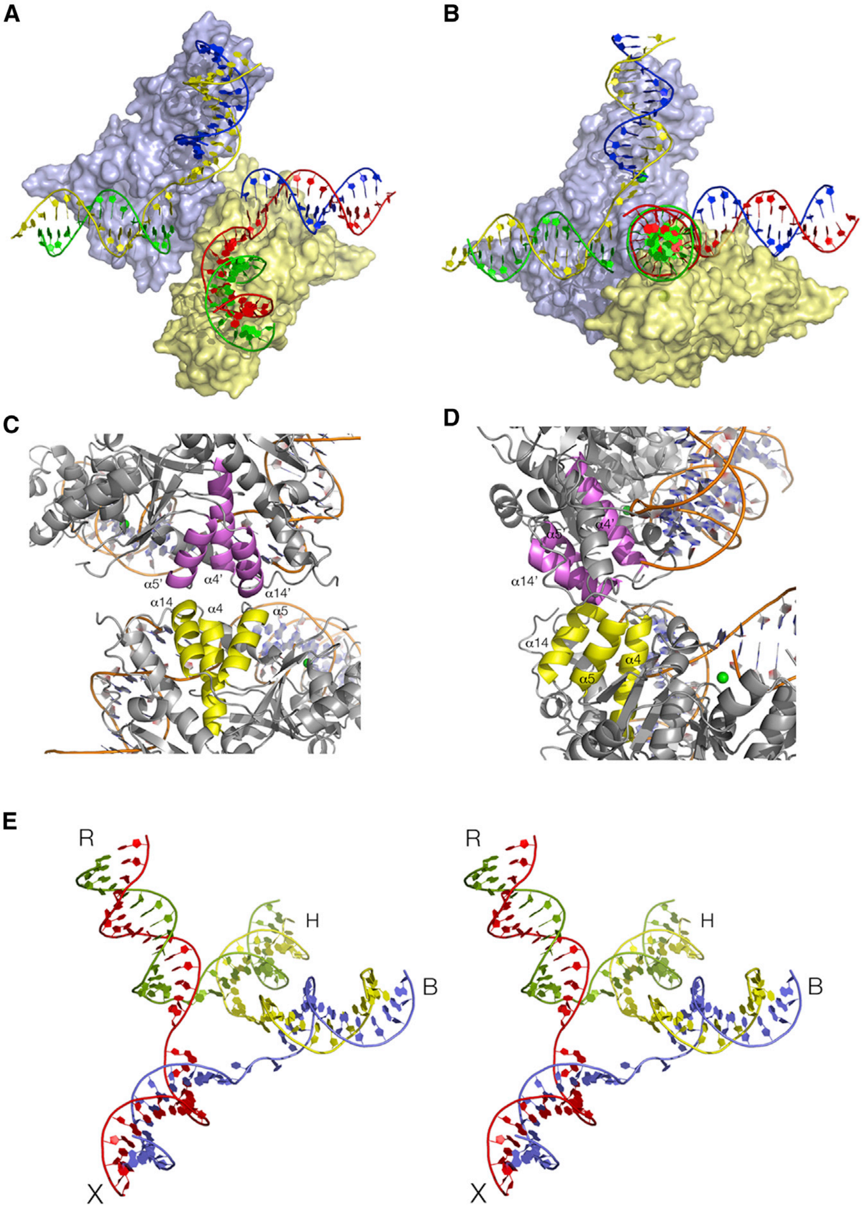

Figure 5. A Dimeric Form of the Complex in the Crystal Lattice

(A and B) Two different views of the complex, with the strands colored to match the expected products of resolution as shown in the scheme in Figure 1A. (A) The view is approximately down the 2-fold axis relating the two cleaved arms (B and R), with the coaxial arms (X and H) lying horizontally across the page. (B) The complex has been rotated around the axis of the coaxial arms so as to view down the axis of one of the cleaved arms (arm R).

(C and D) Two orthogonal views of the dimerization interface, comprising helices α4, α5, and α14 from each monomer (highlighted yellow and magenta).

(E) The strands of the products within the dimeric complex were reconnected requiring only a local change in DNA conformation with opening of base-pairing in the central region. Otherwise, the DNA conformation was completely unaltered. Only the DNA structure of the reconnected junction is displayed in this parallel-eye stereoscopic view. The uncleaved arms H and X are coaxial, with the cleaved B and R arms perpendicular to them and to each other.

Parallel-eye stereoscopic versions of (A)–(C) are provided in Figure S5. A close view of the reconnected junction is shown in Figure S6. See also Movies S2 and S3.

similar mobility to RX, and species BH is expected to be equivalent to RX as the other product species, not observed in the crystal. So once again this requires HX to include 180°. This only leaves the angle BR (i.e., that between the cleaved helices) undetermined. While that species migrates as a slightly less well-defined band, it is evidently similar in mobility to the other 90° species, so these helices should also be close to mutually perpendicular. Thus, the only model that is compatible with all the angular constraints is that shown in Figure 6. This can be visualized by laying the junction in a plane with the arms pointing to the four corners of a square and then lifting the B and R arms (i.e., the helices that are cleaved by CtGEN1) up so that they too become mutually perpendicular. In fact, all the helical arms are mutually perpendicular except for the diagonally opposed arms H and X that must be approximately coaxial. The model emerging from the solution study is in complete agreement with the structure observed for the dimeric complex with two product molecules in the crystal (Figure 5E).

Base Pair Opening in the Complex of a DNA Junction Bound to CtGEN1

In altering the conformation of the junction from the stacked X-structure in the absence of protein, CtGEN1 must disrupt the coaxial stacking of the arms, and more extensive disruption of base-pairing is expected if the protein bound junction has the structure presented in Figure 5. Central distortion of junction structure has been observed in a number of complexes with other junction-resolving enzymes (Déclais et al., 2003; Déclais and Lilley, 2000; White and Lilley, 1997). We have previously used two methods to detect base unstacking and disrupted base-pairing; thymine bases become susceptible to electrophilic attack at the 5,6 double bond by permanganate ion, and 2-aminopurine bases exhibit enhancement of fluorescence.

We studied the reactivity of the thymine bases in the four strands of junction 3. Two versions of the junction were individually radioactively [5′-^{32}P]-labeled on either the h or x strand (that have thymine nucleotides at the point of strand exchange) and reacted with 1 mM $KMnO_4$ for 2 min at 25°C in the presence or absence of an excess of CtGEN1. Reacted thymine nucleotides

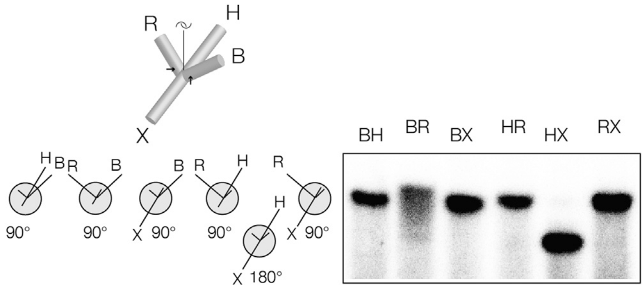

Figure 6. Global Conformation of the DNA Junction Bound by CtGEN1 in Solution Analyzed by Comparative Gel Electrophoresis
Complexes of CtGEN1 bound to the six species of junction 3 with all possible combinations of two long (40 bp) and two short (14 bp) helical arms were electrophoresed in polyacrylamide under non-denaturing conditions. Radioactively [5′-^{32}P]-labeled DNA was visualized by phosphorimaging. Each complex migrates as a single band, and the pattern of migration reflects the geometry of the DNA helical arms in the complex (see text). The position of the cleavage sites in the B and R arms is indicated by arrows on the junction diagram.

were detected by cleavage with piperidine and separation of products by gel electrophoresis and phosphorimaging (Figure 7A). The results show that just a single thymine base of the junction is reactive in the CtGEN1 complex, but not the free DNA, on each of the h and x strands. These two thymine bases are present immediately at the point of strand exchange of the junction, and their reactivity is consistent with a disruption of the structure at the center of the junction on binding CtGEN1.

We analyzed the fluorescent intensity of versions of junction 3 in which a chosen adenine nucleotide was replaced by 2-aminopurine (2-AP). The two adenines either side of the point of strand exchange on the r strand were individually substituted, as were those 3 nt 5′ and 3′ to the point of strand exchange on the b and h strands respectively. The 2-AP substituted junctions were titrated with CtGEN1 in the presence of 1 mM Ca^{2+} ions to prevent cleavage. 2-Aminopurine 3 nt distant from the junction exhibited no significant change in fluorescent intensity on addition of CtGEN1. By contrast, the two 2-AP bases located immediately adjacent to the junction exhibited marked increases in intensity (Figures 7B and 7C). The 2-AP 5′ to the point of strand exchange on the r strand increased to a plateau level reflecting an ~40-fold enhancement of fluorescence, while that 3′ to the junction increased 10-fold. An increase of such magnitude indicates a major opening of the junction, probably involving local loss of base-pairing, although clearly this effect does not extend as far as the third base pair. We have previously observed a similar level of disruption of a junction by the yeast mitochondrial enzyme Cce1 (Déclais and Lilley, 2000).

We repeated the same analysis on equivalent DNA species constructed to represent the product of junction resolution. Although 2-AP located at the r-1 position exhibited an increase in fluorescence intensity on addition of CtGEN1, this was only half that observed for the complete junction (Figure 7C). This was confirmed by activating the CtGEN1 in the complex with the junctions by addition of an excess of Mg^{2+} ions, whereupon the 2-AP fluorescence intensity reduced by 50% (Figure S7).

DISCUSSION

The structure of CtGEN1 clearly reveals its heritage (Figures 1 and 2). Its protein architecture is closely related to those of the other FEN1-XPG family members, including its active site, which is that of a standard two-metal-ion-mechanism nuclease (Figure 4). A flap can be considered to be half of a four-way junction, and so it might be anticipated that dimerization of a FEN-type domain could generate a junction-resolving enzyme. Our structural studies show that CtGEN1 is effectively an elaborated form of FEN1 that has substantially dispensed with the helical arch that is not required for the selection of a single-stranded section. Instead, this region of CtGEN1 is probably involved in recognition of the central structure of the junction, and in part contributes to the dimerization domain, while a new C-terminal section makes additional contacts to the DNA so as to increase the affinity and selectivity for the structure of the junction once dimerization has occurred.

In our experiments, we crystallized a wild-type sequence in the presence of Mg^{2+} ions. Under these conditions, CtGEN1 is fully active, and the species crystallized is the product of resolution cleavage. The crystallization process has selected a single product of enzymatic cleavage, containing the 30 nt r strand from the junction and the 3′ half of the h strand and the 5′ half of the x strand, i.e., the product with R and X arms. The expected one nucleotide 3′ overhang on the x strand is not visible in the electron density, presumably because it is too mobile. Similarly, the 5′ nucleotide of the x strand is unpaired and not visible in the electron density.

Although the functional unit observed in the crystal corresponds to one of the products of resolution (Figure 1), protein-protein interaction between the CtGEN1 monomers in the crystal lattice generates a structure that is clearly related to that of the dimeric enzyme bound to a four-way junction (Figure 5). The two DNA species are held by the proteins so that the uncleaved arms (these would be the H and X arms of the complete junction) are close to coaxial, while cleaved arms (B and R in the junction) are mutually perpendicular and perpendicular to the H-X axis. This is exactly the disposition of arms that was deduced for the junction in solution from the comparative gel electrophoresis experiments (Figure 6). We found that it was possible to reconnect the DNA strands in the complex observed in the crystal to generate an intact four-way junction without altering the disposition of the arms (Figure 5E), but this required a degree of helical opening at the junction center. This is fully in agreement with the observation of enhanced chemical reactivity and 2-aminopurine fluorescence on addition of CtGEN1 to a four-way junction (Figure 7). The opening of the four-way junction by CtGEN1 is similar to that induced by the majority of junction-resolving enzymes (Déclais and Lilley, 2008).

The dimerization interface observed in the crystallized complex primarily comprises the helices α4, α5, and α14 and associated loops (Figures 5C and 5D). Dimerization involves a relatively small contact area of 530 Å^2, consistent with a low tendency of the protein to dimerize in free solution (Freeman et al., 2014). Binding to a DNA junction is strongly cooperative, with a Hill coefficient >3 (Table S1). These observations suggest that CtGEN1 exists in solution primarily in monomeric form and dimerizes on

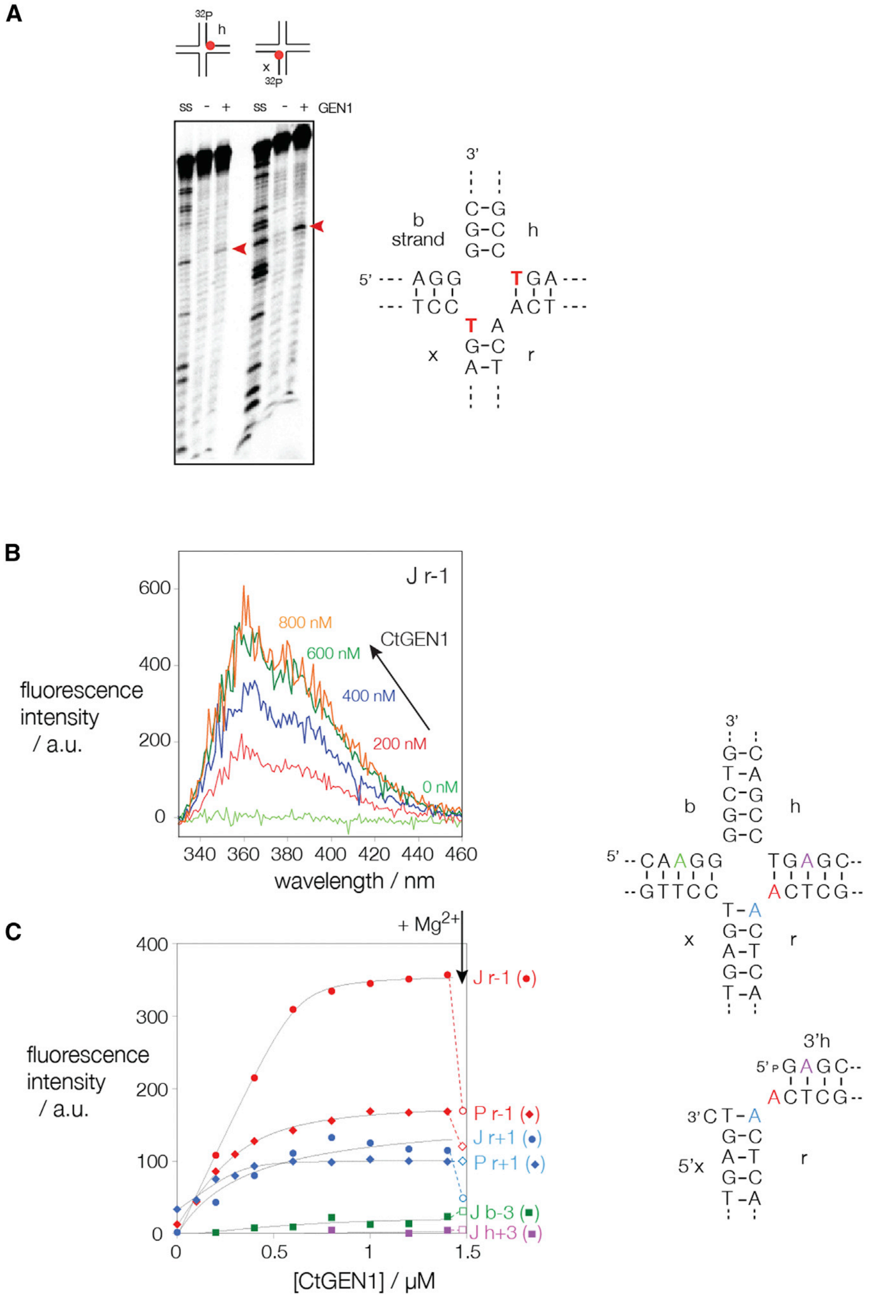

Figure 7. DNA Opening at the Center of the CtGEN1 Complex Studied in Solution

(A) Reactivity of thymine bases to permanganate. Junction 3 was radioactively [5′-^{32}P]-labeled on the h or x strands. Single-stranded oligonucleotide (*ss*), protein-free junction (–), and junction in complex with CtGEN1 (+) were incubated with 1 mM $KMnO_4$ for 2 min. After termination of the reaction the DNA was cleaved with 1 M piperidine, and the DNA products were separated by gel electrophoresis under denaturing conditions and visualized by phosphorimaging. The arrowed bands indicating sites of enhanced reactivity in the complex correspond to the thymine bases highlighted in red in the sequence of the junction (right). Note that these lie at the point of strand exchange in the junction.

(B and C) Enhancement of 2-aminopurine fluorescence in the junction (right, upper) and product of resolution (right, lower). Junction and product were prepared with individual adenine nucleotides replaced by 2-aminopurine at the positions indicated in color. The fluorescence emission spectrum of the junction with 2-aminopurine on the r strand 1 nt 5′ to the point of strand exchange as a function of CtGEN1 concentration is shown in (B). Note the enhancement of intensity as the stoichiometry of CtGEN1 increases. The fluorescence intensity for all the constructs is plotted as a function of CtGEN1 concentration in (C). The data are labeled J for junction species and P for product species with the position of substitution appended. Note that the species substituted at the nucleotides adjacent to the point of strand exchange exhibit the strong enhancement of fluorescence on enzyme binding. Titrations were performed in the presence of 1 mM $CaCl_2$ so that the CtGEN1 was inactive. After the last addition of CtGEN1, an excess of $MgCl_2$ was added to activate the enzyme leading to a reduction in the fluorescence intensity for the junction species (open symbols connected by broken lines). Note that for the Jr-1 species, the resulting intensity is similar to that for the product (Pr-1).

See also Figure S7.

binding to the junction. This provides an opportunity for regulation of activity that is not possible in resolving enzymes from lower organisms that exist in solution in dimeric form.

Binding of active monomeric CtGEN1 to a junction could potentially generate undesirable unilateral cleavage of a four-way junction prior to dimer formation. We postulate that monomeric CtGEN1 activity is suppressed by a partially disordered active site, providing a failsafe mechanism. The loop between β3 and α5 where the helical arch in FEN1 is located is disordered in the product complex, yet contains a number of basic residues that are likely to play a role in both branch point distortion and the cleavage reaction. The disordered region is in close proximity both to the dimer interface and to the reconnected strands in our model of the intact four-way junction, suggesting that this part of the active site only becomes structured when a CtGEN1 dimer is bound to the intact junction, thereby activating the enzyme for cleavage. According to this hypothesis, CtGEN1 monomer bound to a four-way junction would be relatively inactive, thus minimizing unilateral cleavage of four-way junctions and preventing more promiscuous activity on flaps and other kinds of junctions. We are investigating these possibilities experimentally.

The structure of the DNA product bound to CtGEN1, and the reduction in 2-aminopurine fluorescence on cleavage by CtGEN1, indicates that the DNA structure at the center of the intact junction is more open than the product, where the base

adjacent to the cleavage site is paired. Moreover, the position of the 5′-terminal phosphate relative to the two metal ions in the cleavage site in the product complex is not suitable for in-line attack by a metal-bound water molecule, so the geometry in the active site must have rearranged after cleavage. It is also very probable that the metal ions have moved from their positions prior to the cleavage reaction. This relaxation is in contrast with what was observed in the post-cleavage complexes of FEN1 and EXO1, where the scissile phosphate remains bound to the metal ions, and the adjacent base is unpaired. This could explain our observation that in the resolution of a junction, the second cleavage reaction occurs ten times faster than the first (Freeman et al., 2014). If relaxation of the DNA structure following first strand cleavage leads to a readjustment of the structure of the complex so that the second strand is better accommodated into the active site, this could lead to an acceleration of the hydrolytic reaction. This would increase the probability that bilateral cleavage will occur during the lifetime of the enzyme-junction complex and thus ensure a productive resolution of the junction, with a lower probability of release of a semi-resolved junction.

The structure presented here reveals how CtGEN1 is specific for the structure of a four-way DNA junction and suggests how it ensures that a productive resolution results from the interaction.

EXPERIMENTAL PROCEDURES

Full experimental details are presented in the Supplemental Information.

Sample Preparation and Purification

C. thermophilum GEN1 1-487 with a C-terminal six-histidine tag was expressed in *E. coli* BL21(DE3) RIL (Stratagene). CtGEN1 was purified by Ni-NTA affinity, heparin, gel filtration, and ion exchange chromatography. Purified CtGEN1 migrated as a single band on an overloaded polyacrylamide gel in the presence of SDS.

Crystallization, Data Collection, and Structure Determination

Purified CtGEN1 was mixed with a four-way DNA junction based on junction 3 (Duckett et al., 1988) and comprising 15 bp in each helical arm (100 μM each of CtGEN1 monomer and DNA junction). Equal volumes of DNA and protein were mixed in a final concentration of 100 mM HEPES (pH 7.5), 2 mM $MgCl_2$, 20% PEG10000, and incubated with the same buffer using hanging drop vapor diffusion at 7°C. Crystals were soaked in cryo-protectant, dehydrated by vapor diffusion equilibration, and stored under liquid nitrogen. Initial phases were acquired from the SAD data by locating the eight selenium atoms with Autosol in the PHENIX suite (Adams et al., 2010). The initial model was generated automatically by PHENIX autobuild wizard and then applied to the native datasets by molecular replacement using Phaser (McCoy et al., 2007). The model was adjusted manually and subjected to several rounds of adjustment and optimization. Datasets for the complexes with native CtGEN1 in Mg^{2+} and Mn^{2+} and the Se-methionine-substituted CtGEN1 were obtained using synchrotron X-radiation at a resolution of 2.5, 2.6, and 3.15 Å, respectively.

Analysis of Cleavage and Binding Affinity with a Four-Way DNA Junction Using Point Mutants of CtGEN1

Active site residues of CtGEN1 were individually converted into alanine by PCR. Rates of cleavage of junction 3 by wild-type and mutant CtGEN1 were measured under single-turnover conditions. The fraction of DNA cleaved at time t (F_t) was fitted to:

$$F_t = F_f.(I - exp(-k_c t)). \qquad \text{(Equation 1)}$$

Binding affinity was measured by electrophoretic retardation analysis. Data were analyzed as fraction DNA bound (f_b) versus protein concentration and fitted to:

$$f_b = 1/\left(1 + (K_d/P_t)^n\right), \qquad \text{(Equation 2)}$$

where K_d is the dissociation constant, P_t is the total protein monomer concentration.

Comparative Gel Electrophoresis

The six possible DNA junctions with two 40 bp and two 14 bp arms were incubated with 100 nM CtGEN1, loaded onto a polyacrylamide gel in the presence of 2 mM $CaCl_2$, and run under native conditions.

Permanganate Probing of the DNA Junction

Junction 3 with and without CtGEN1 was reacted with 1 mM $KMnO_4$ for 2 min at 25°C and site-specifically cleaved by incubation with 1 M piperidine at 95°C for 30 min. The products were separated by gel electrophoresis under denaturing conditions.

Fluorescence Spectroscopy

Junction 3 and its corresponding r-strand resolution cleavage product were prepared with adenine nucleotides substituted by 2-aminopurine at selected single positions. Steady-state fluorescence emission spectra were recorded between 330 nm and 460 nm in 1 nm intervals with excitation at 315 nm. Spectra were integrated between 370 and 410 nm to calculate binding curves.

ACCESSION NUMBERS

The accession number for the complex in Mg^{2+} ions, modeled as two DNA duplexes with two CtGEN1 proteins overlaid at 50% occupancy reported in this paper, is PDB: 5CO8. The accession number for the complex after soaking in Mn^{2+} ions, modeled as one CtGEN1 protein with an R-stem DNA duplex, is PDB: 5CNQ.

SUPPLEMENTAL INFORMATION

Supplemental Information includes Supplemental Experimental Procedures, seven figures, one table, and three movies and can be found with this article online at http://dx.doi.org/10.1016/j.celrep.2015.11.042.

ACKNOWLEDGMENTS

We thank Saira Ashraf for DNA synthesis and Prof. Bill Hunter for discussion and acknowledge financial support from Cancer Research UK (D.M.J.L. laboratory) and the Wellcome Trust (A.G.). We thank both Diamond and the European Synchrotron Radiation Facility for synchrotron time and the Wellcome Trust for the in-house diffractometer.

Received: July 30, 2015
Revised: October 4, 2015
Accepted: November 11, 2015
Published: December 10, 2015

REFERENCES

Adams, P.D., Afonine, P.V., Bunkóczi, G., Chen, V.B., Davis, I.W., Echols, N., Headd, J.J., Hung, L.W., Kapral, G.J., Grosse-Kunstleve, R.W., et al. (2010). PHENIX: a comprehensive Python-based system for macromolecular structure solution. Acta Crystallogr. D Biol. Crystallogr. *66*, 213–221.

Agostinho, A., Meier, B., Sonneville, R., Jagut, M., Woglar, A., Blow, J., Jantsch, V., and Gartner, A. (2013). Combinatorial regulation of meiotic holliday junction resolution in *C. elegans* by HIM-6 (BLM) helicase, SLX-4, and the SLX-1, MUS-81 and XPF-1 nucleases. PLoS Genet. *9*, e1003591.

Andersen, S.L., Bergstralh, D.T., Kohl, K.P., LaRocque, J.R., Moore, C.B., and Sekelsky, J. (2009). Drosophila MUS312 and the vertebrate ortholog

BTBD12 interact with DNA structure-specific endonucleases in DNA repair and recombination. Mol. Cell *35*, 128–135.

Bailly, A.P., Freeman, A., Hall, J., Déclais, A.C., Alpi, A., Lilley, D.M., Ahmed, S., and Gartner, A. (2010). The *Caenorhabditis elegans* homolog of Gen1/Yen1 resolvases links DNA damage signaling to DNA double-strand break repair. PLoS Genet. *6*, e1001025.

Cejka, P., Plank, J.L., Bachrati, C.Z., Hickson, I.D., and Kowalczykowski, S.C. (2010). Rmi1 stimulates decatenation of double Holliday junctions during dissolution by Sgs1-Top3. Nat. Struct. Mol. Biol. *17*, 1377–1382.

Ceska, T.A., Sayers, J.R., Stier, G., and Suck, D. (1996). A helical arch allowing single-stranded DNA to thread through T5 5′-exonuclease. Nature *382*, 90–93.

Constantinou, A., Davies, A.A., and West, S.C. (2001). Branch migration and Holliday junction resolution catalyzed by activities from mammalian cells. Cell *104*, 259–268.

Constantinou, A., Chen, X.-B., McGowan, C.H., and West, S.C. (2002). Holliday junction resolution in human cells: two junction endonucleases with distinct substrate specificities. EMBO J. *21*, 5577–5585.

Déclais, A.C., and Lilley, D.M.J. (2008). New insight into the recognition of branched DNA structure by junction-resolving enzymes. Curr. Opin. Struct. Biol. *18*, 86–95.

Déclais, A.-C., and Lilley, D.M.J. (2000). Extensive central disruption of a four-way junction on binding CCE1 resolving enzyme. J. Mol. Biol. *296*, 421–433.

Déclais, A.-C., Hadden, J., Phillips, S.E.V., and Lilley, D.M.J. (2001). The active site of the junction-resolving enzyme T7 endonuclease I. J. Mol. Biol. *307*, 1145–1158.

Déclais, A.-C., Fogg, J.M., Freeman, A.D., Coste, F., Hadden, J.M., Phillips, S.E.V., and Lilley, D.M.J. (2003). The complex between a four-way DNA junction and T7 endonuclease I. EMBO J. *22*, 1398–1409.

Duckett, D.R., Murchie, A.I.H., Diekmann, S., von Kitzing, E., Kemper, B., and Lilley, D.M.J. (1988). The structure of the Holliday junction, and its resolution. Cell *55*, 79–89.

Duckett, D.R., Panis, M.J., and Lilley, D.M.J. (1995). Binding of the junction-resolving enzyme bacteriophage T7 endonuclease I to DNA: separation of binding and catalysis by mutation. J. Mol. Biol. *246*, 95–107.

Elborough, K.M., and West, S.C. (1990). Resolution of synthetic Holliday junctions in DNA by an endonuclease activity from calf thymus. EMBO J. *9*, 2931–2936.

Ellis, N.A., Groden, J., Ye, T.Z., Straughen, J., Lennon, D.J., Ciocci, S., Proytcheva, M., and German, J. (1995). The Bloom's syndrome gene product is homologous to RecQ helicases. Cell *83*, 655–666.

Fekairi, S., Scaglione, S., Chahwan, C., Taylor, E.R., Tissier, A., Coulon, S., Dong, M.Q., Ruse, C., Yates, J.R., 3rd, Russell, P., et al. (2009). Human SLX4 is a Holliday junction resolvase subunit that binds multiple DNA repair/recombination endonucleases. Cell *138*, 78–89.

Freeman, A.D.J., Liu, Y., Déclais, A.-C., Gartner, A., and Lilley, D.M.J. (2014). GEN1 from a thermophilic fungus is functionally closely similar to non-eukaryotic junction-resolving enzymes. J. Mol. Biol. *426*, 3946–3959.

Garner, E., Kim, Y., Lach, F.P., Kottemann, M.C., and Smogorzewska, A. (2013). Human GEN1 and the SLX4-associated nucleases MUS81 and SLX1 are essential for the resolution of replication-induced Holliday junctions. Cell Rep. *5*, 207–215.

Giraud-Panis, M.-J.E., and Lilley, D.M.J. (1998). Structural recognition and distortion by the DNA junction-resolving enzyme RusA. J. Mol. Biol. *278*, 117–133.

Grasby, J.A., Finger, L.D., Tsutakawa, S.E., Atack, J.M., and Tainer, J.A. (2012). Unpairing and gating: sequence-independent substrate recognition by FEN superfamily nucleases. Trends Biochem. Sci. *37*, 74–84.

Holliday, R. (1964). A mechanism for gene conversion in fungi. Genet. Res. *89*, 285–307.

Holm, L., and Rosenström, P. (2010). Dali server: conservation mapping in 3D. Nucleic Acids Res. *38*, W545–W549.

Hosfield, D.J., Mol, C.D., Shen, B., and Tainer, J.A. (1998). Structure of the DNA repair and replication endonuclease and exonuclease FEN-1: coupling DNA and PCNA binding to FEN-1 activity. Cell *95*, 135–146.

Ip, S.C., Rass, U., Blanco, M.G., Flynn, H.R., Skehel, J.M., and West, S.C. (2008). Identification of Holliday junction resolvases from humans and yeast. Nature *456*, 357–361.

Lilley, D.M.J. (2008). Analysis of branched nucleic acid structure using comparative gel electrophoresis. Q. Rev. Biophys. *41*, 1–39.

Lorenz, A., West, S.C., and Whitby, M.C. (2010). The human Holliday junction resolvase GEN1 rescues the meiotic phenotype of a *Schizosaccharomyces pombe* mus81 mutant. Nucleic Acids Res. *38*, 1866–1873.

Matos, J., Blanco, M.G., Maslen, S., Skehel, J.M., and West, S.C. (2011). Regulatory control of the resolution of DNA recombination intermediates during meiosis and mitosis. Cell *147*, 158–172.

McCoy, A.J., Grosse-Kunstleve, R.W., Adams, P.D., Winn, M.D., Storoni, L.C., and Read, R.J. (2007). Phaser crystallographic software. J. Appl. Cryst. *40*, 658–674.

Miętus, M., Nowak, E., Jaciuk, M., Kustosz, P., Studnicka, J., and Nowotny, M. (2014). Crystal structure of the catalytic core of Rad2: insights into the mechanism of substrate binding. Nucleic Acids Res. *42*, 10762–10775.

Muñoz, I.M., Hain, K., Déclais, A.-C., Gardiner, M., Toh, G.W., Sanchez-Pulido, L., Heuckmann, J.M., Toth, R., Macartney, T., Eppink, B., et al. (2009). Coordination of structure-specific nucleases by human SLX4/BTBD12 is required for DNA repair. Mol. Cell *35*, 116–127.

Orans, J., McSweeney, E.A., Iyer, R.R., Hast, M.A., Hellinga, H.W., Modrich, P., and Beese, L.S. (2011). Structures of human exonuclease 1 DNA complexes suggest a unified mechanism for nuclease family. Cell *145*, 212–223.

Pöhler, J.R.G., Giraud-Panis, M.-J.E., and Lilley, D.M.J. (1996). T4 endonuclease VII selects and alters the structure of the four-way DNA junction; binding of a resolution-defective mutant enzyme. J. Mol. Biol. *260*, 678–696.

Rass, U., Compton, S.A., Matos, J., Singleton, M.R., Ip, S.C., Blanco, M.G., Griffith, J.D., and West, S.C. (2010). Mechanism of Holliday junction resolution by the human GEN1 protein. Genes Dev. *24*, 1559–1569.

Steitz, T.A., and Steitz, J.A. (1993). A general two-metal-ion mechanism for catalytic RNA. Proc. Natl. Acad. Sci. USA *90*, 6498–6502.

Svendsen, J.M., Smogorzewska, A., Sowa, M.E., O'Connell, B.C., Gygi, S.P., Elledge, S.J., and Harper, J.W. (2009). Mammalian BTBD12/SLX4 assembles a Holliday junction resolvase and is required for DNA repair. Cell *138*, 63–77.

Syson, K., Tomlinson, C., Chapados, B.R., Sayers, J.R., Tainer, J.A., Williams, N.H., and Grasby, J.A. (2008). Three metal ions participate in the reaction catalyzed by T5 flap endonuclease. J. Biol. Chem. *283*, 28741–28746.

Tsutakawa, S.E., Classen, S., Chapados, B.R., Arvai, A.S., Finger, L.D., Guenther, G., Tomlinson, C.G., Thompson, P., Sarker, A.H., Shen, B., et al. (2011). Human flap endonuclease structures, DNA double-base flipping, and a unified understanding of the FEN1 superfamily. Cell *145*, 198–211.

White, M.F., and Lilley, D.M.J. (1996). The structure-selectivity and sequence-preference of the junction-resolving enzyme CCE1 of *Saccharomyces cerevisiae*. J. Mol. Biol. *257*, 330–341.

White, M.F., and Lilley, D.M.J. (1997). The resolving enzyme CCE1 of yeast opens the structure of the four-way DNA junction. J. Mol. Biol. *266*, 122–134.

Wu, L., and Hickson, I.D. (2003). The Bloom's syndrome helicase suppresses crossing over during homologous recombination. Nature *426*, 870–874.

Cell Reports

Supplemental Information

Crystal Structure of a Eukaryotic GEN1 Resolving Enzyme Bound to DNA

Yijin Liu, Alasdair D.J. Freeman, Anne-Cécile Déclais, Timothy J. Wilson, Anton Gartner, and David M.J. Lilley

SUPPLEMENTARY FIGURES

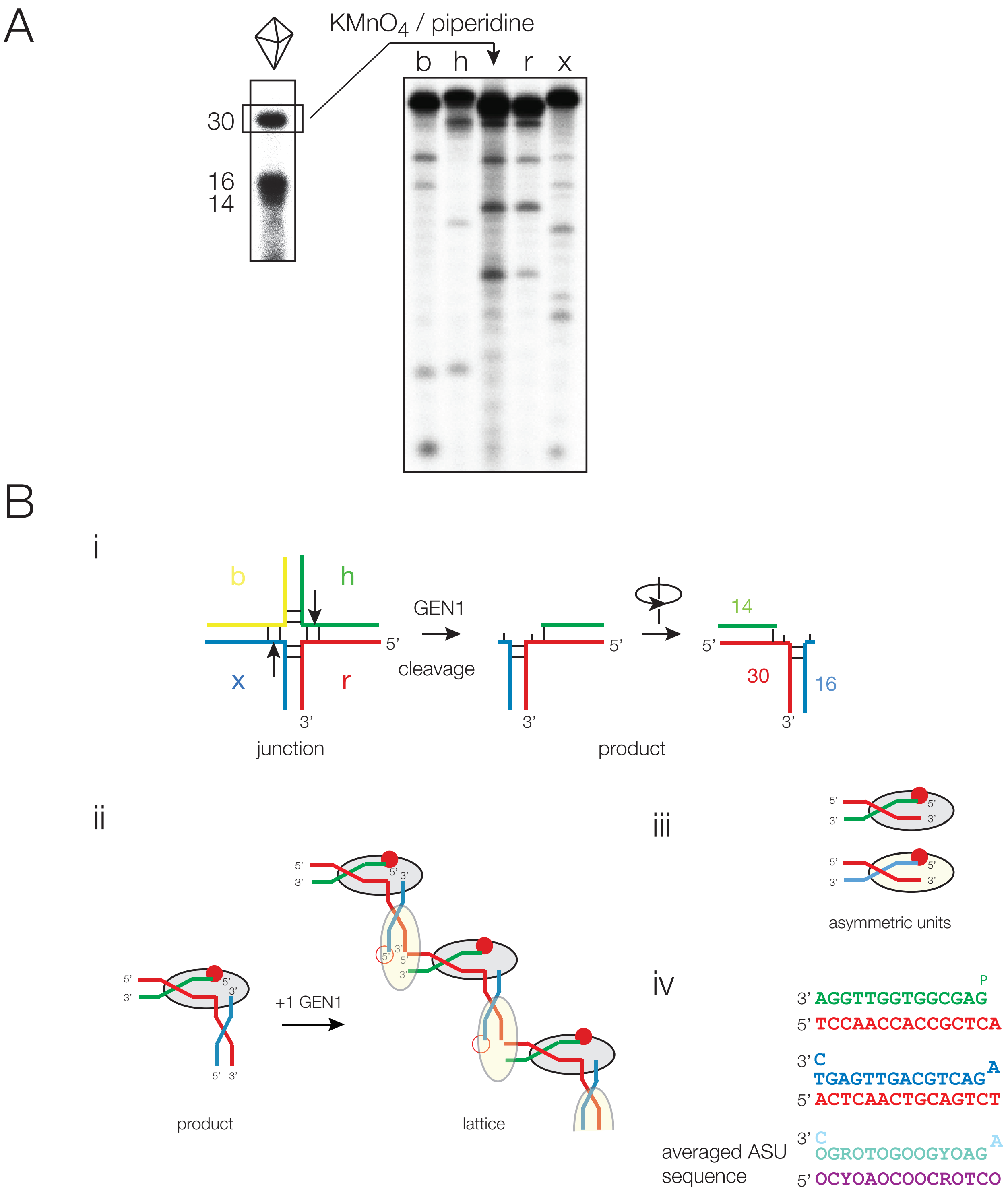

Figure S1. The Contents of the Crystal, and the Probable Origin of the Observed Lattice; Related to Figure 1.
A. Analysis of DNA in crystals used to collect X-ray diffraction data. Crystals previously used for data collection at the synchrotron were resuspended in 10 μl 10 mM Hepes (pH 7.5), 50 mM KCl, 1 mM $CaCl_2$ and incubated with 20 μg of proteinase K for 16 h at 37°C. The protease was inactivated by incubation with 1 mM PMSF for 20 min, and DNA denatured by heating. The DNA was radioactively [5'-^{32}P]-labeled using T4 polynucleotide kinase and [γ-^{32}P] ATP. The DNA was analyzed by electrophoresis in a 20% (19:1) polyacrylamide in the presence of 8M urea (*left*). After phosphorimaging, two bands of radioactive DNA are seen. The upper one corresponds to 30 nt, while the lower one is

interpreted in terms of the presence of two species of 16 and 14 nt. DNA was recovered from the upper band by electroelution. This was reacted with $KMnO_4$, and followed by incubation with piperidine to cleave the backbone at the positions of modified thymine nucleotides as detailed in the Material and Methods. Each strand of the junction used in crystallization was also independently labeled and subjected to $KMnO_4$ / piperidine reaction. All five samples were electrophoresed in a 20% (19:1) polyacrylamide gel in buffer containing 8M urea (*right*). It is clear that the mobility of the full-length strand and the pattern of bands of greater mobility (corresponding to cleavage at permanganate-modified thymine nucleotides) observed in the 30 nt oligonucleotide extracted from the crystal closely matches that of the r strand. By contrast, bands unique to the b strand are not visible in this gel. Tracks L to R : b strand, h strand, extracted 30 nt oligonucleotide, r strand and x strand.

B. The probable origin of the CtGEN1-DNA complexes observed in the crystal. All available evidence indicates that the crystal contains a complex of a monomer of CtGEN1 with the product of resolution cleavage that predominantly contains the r strand.

i) The major cleavage of the four-way junction (with four arms each of 15 bp, shown left) by CtGEN1 occurs at the sites in the h and x strands shown by the arrows (Freeman et al., 2014), to produce the r-strand product (center). This is also shown rotated around the vertical axis (right). The overhanging nucleotides are not shown in the remaining diagrams.

ii) Schematic of the complex of the product DNA bound to one molecule of CtGEN1 (left). CtGEN1 is depicted as the grey ellipse, with the active site indicated by the red spot. Binding of a second molecule of GEN1 (colored yellow) in a quasi-equivalent manner with the free ends (i.e. the 3' end of the h strand and the 5' end of the x strand) generates the end to end chain of complexes observed in the crystal lattice. However the parallel chains that run through the crystal lattice (see supplementary movie S2) are not all in register; the products in one strand could align with those of an adjacent strand, or they could be offset such that the h strands in one chain align with the x strands of an adjacent strand. This is because all of the contacts within the lattice are protein mediated, and GEN1 binds each half of the product in an equivalent manner. This lack of alignment results in the averaging of the DNA sequences in the electron density map.

iii) This generates two kinds of complex as the asymmetric unit, each containing a different half of the product nicked duplex.

iv) The quasi-equivalence in the asymmetric unit generates an averaged nucleotide sequence. These sequences are written with the top strand running 3' to 5' in each case. The sequence of the DNA cannot be read from the electron density map in a straightforward way, but can be interpreted in terms of an average of the h and x sequences. R = purine, Y = pyrimidine, O = averaged purine/pyrimidine. The 3'-cytosine and 5'-adenosine of the x strand should have occupancies of 0.5, but are not observed. There is space for them to be present, but they are probably too mobile within the structure to be observable.

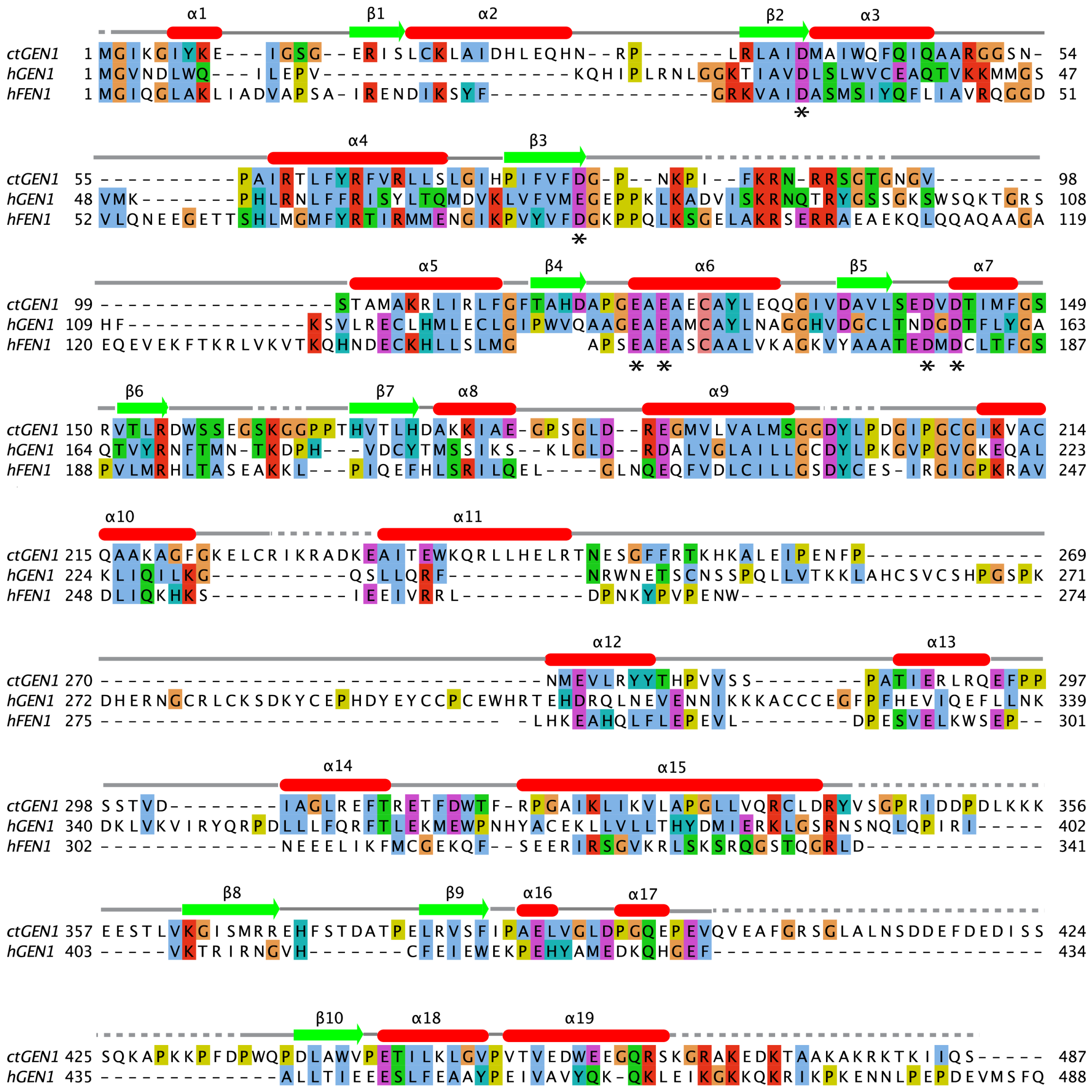

Figure S2. An Alignment of CtGEN1 (ctGEN1), Human GEN1 (hGEN1) and Human FEN1 (hFEN1) with the Secondary Structure Elements of CtGEN1; Related to Figure 2. Secondary structure is shown as α-helix (red) and β-sheet (green). Regions not observed in the electron density maps are shown by a broken line. Active site acidic residues are marked by an asterisk.

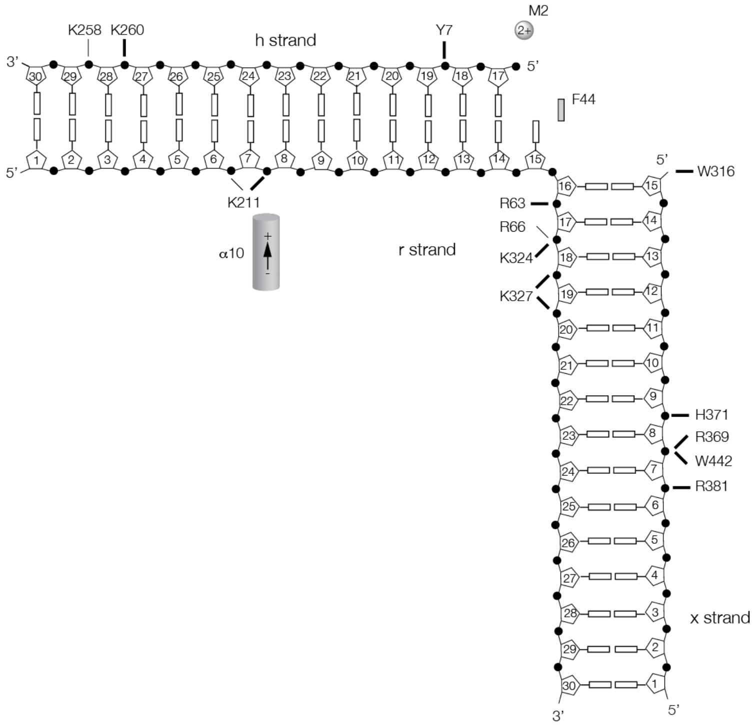

Figure S3. A Map of DNA-Protein Contacts in the Functional Unit of the CtGEN1 Complex; Related to Figure 3.
The cylinder shows the helix dipole of α10, with its N-terminus (positive pole) directed at a phosphate group in the cleaved DNA helix.

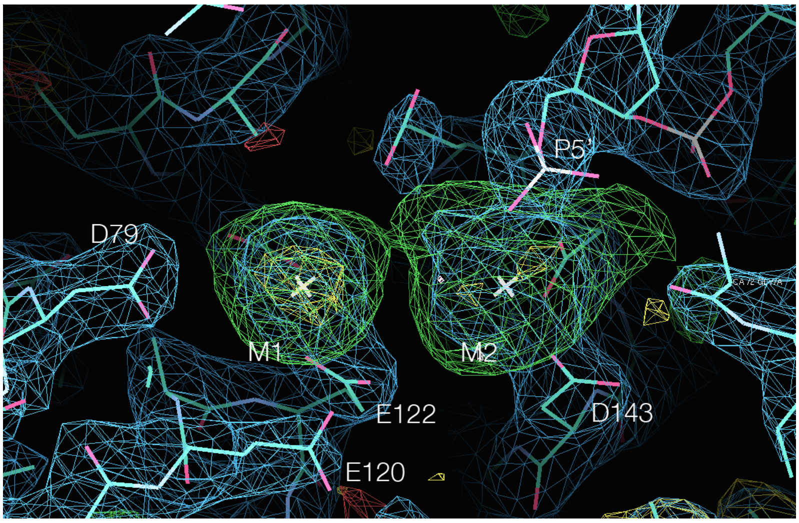

Figure S4. Electron Density Map of the Active Site for the Mn^{2+}-Soaked Crystals using Anomalous Dispersion by Mn^{2+} Ions; Related to Figure 4.
The data were collected at $\lambda = 0.91$ Å in the high energy remote area of the manganese K absorption edge to prevent protein crystal radiation damage. *Blue* contouring : $2\mathbf{F}_o$-$\mathbf{F}_c$ map contoured at 1.5 σ. *Green* contouring : $\mathbf{F}_o$-$\mathbf{F}_c$ simulated annealing omit map contoured at 3 σ showing density consistent with the presence of two metal ions. *Yellow* contouring: Anomalous dispersion map for Mn^{2+} ions contoured at 3 σ. These data indicate that during the crystal soaking the Mg^{2+} ion is mostly retained in the M2 position, but the M1 site becomes predominantly occupied by a Mn^{2+} ion.

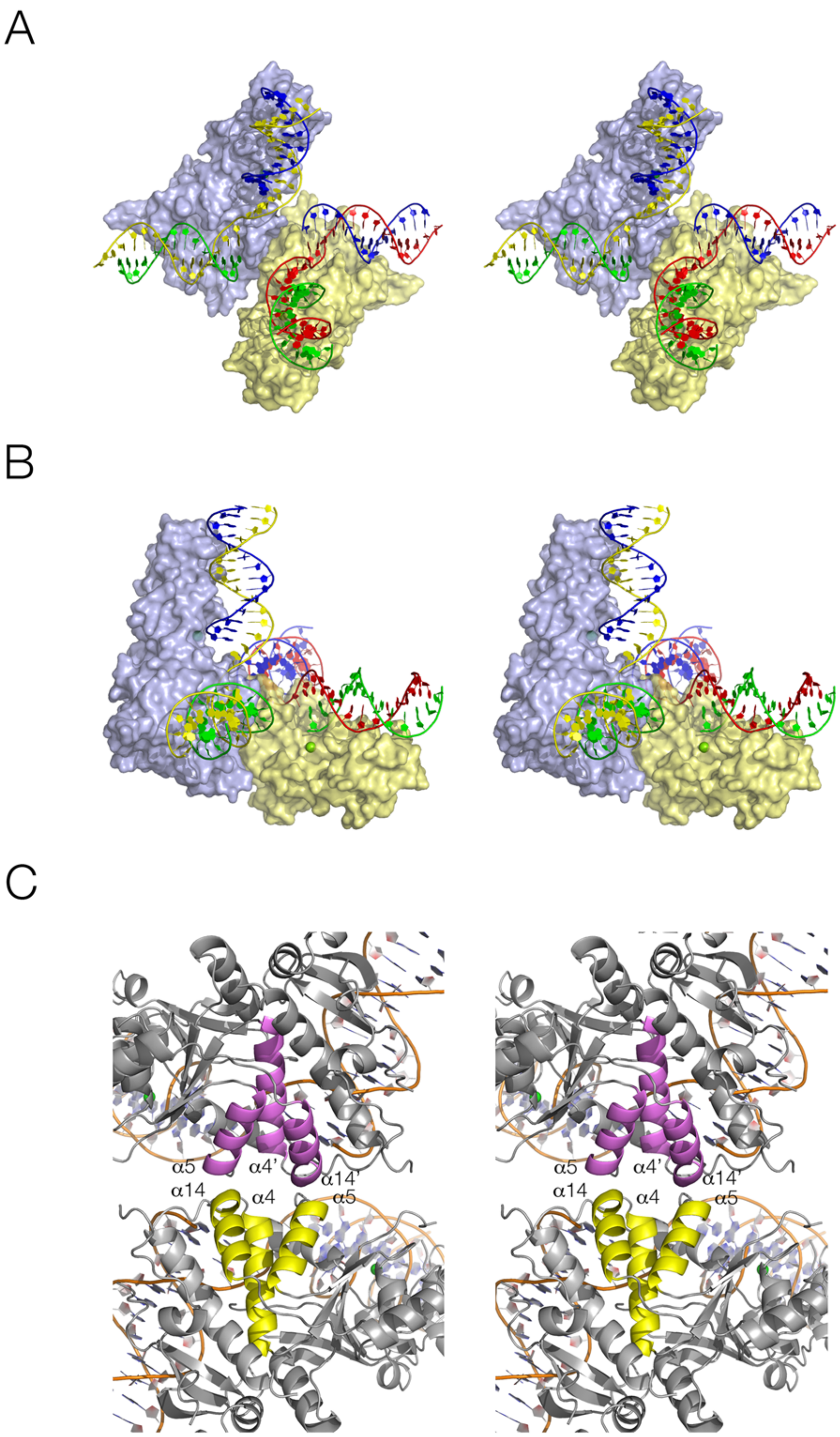

Figure S5. Parallel-Eye Stereoscopic Views of the Dimeric Form of the Complex Found in the Crystal Lattice; Related to Figure 5. **A, B**. Two views of the complex, with the strands colored to match the expected products of resolution as shown in the scheme in Figure S1Bi.

C. Close view of the dimerization interface, comprising helices α4, α5 and α14 from each monomer (highlighted yellow and magenta).

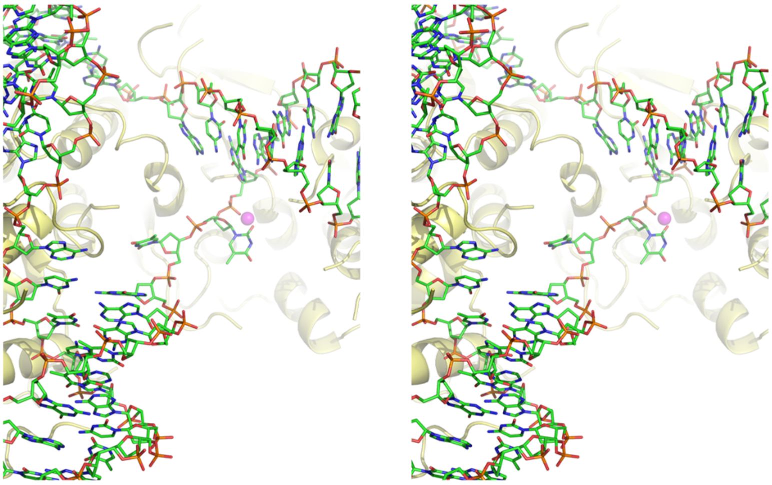

Figure S6. Close View of the Reconnected Junction; Related to Figure 5.
Parallel-eye stereoscopic close view of the sections that have been reconnected to form the fully-intact junction.

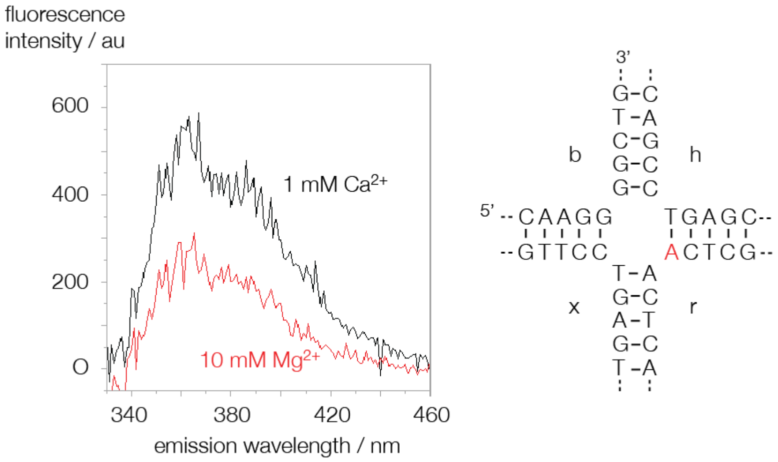

Figure S7. Fluorescence Spectra of 2-Aminopurine at the r-1 Position of the Junction as a Function Enzymatic Cleavage; Related to Figure 7.
Fluorescence emission spectra of junction 3 with adenine substituted by 2-aminopurine at the r-1 position (colored red) complexed with CtGEN as a function of the nature of the divalent cation. The spectrum was recorded in 1 mM Ca^{2+} ions (black), where the enzyme is inactive, and after the addition of 10 mM Mg^{2+} ions to allow enzymatic cleavage to occur.

SUPPLEMENTARY MOVIES

Movie S1. The Relationship between the Asymmetric Unit, the Functional Unit and the Lattice. Related to Figure 1.
Scene 1 : The single functional unit, corresponding to the product of GEN1 cleavage. A single GEN1 monomer (blue) binds two helices connected by the continuity of the red strand. The strands are colored as in Figure 1.
Scene 2 : Two more functional units are added, directly repeated head-to-tail , as found in the crystal lattice.
Scene 3 : Two additional GEN1 monomers (yellow) bind to connect the functional units. These then rotate to show how they are bound on opposite faces of the DNA helices.
Scene 4. The blue GEN1 monomers are hidden to show how the additional yellow monomers are bound in an equivalent manner to the first blue monomers. Within these complexes the two DNA helices are not connected by a central phosphate on the red strand.

Movie S2. The Presence of a Dimeric GEN1 Complex within the Crystal Lattice. Related to Figure 5.
Scene 1 : DNA molecules in the crystal lattice, beginning by viewing down the three-fold axis. The three linear chains of products (like that shown in Movie S1) are colored red, blue and green, and form parallel chains through the lattice. The lattice is then rotated sequentially about orthogonal axes.
Scene 2 : most of the molecules become grey, with just two product molecules left colored red and green. The view now zooms into these, and the remaining molecules fade out.
Scene 3 : The two DNA products juxtaposed in the lattice are rotated, and recolored to match the coloring of the strands of the product used in Figure 1A.
Scene 4 : Two bound CtGEN1 molecules now become visible, forming a dimeric complex interacting via a protein-protein interface.

Movie S3. The Dimer Complex, Closely Related to a Four-way Junction bound to two GEN1 Monomers. Related to Figure 5.
Scene 1 : The dimeric complex of CtGEN1 is shown in cartoon form. This rotates and switches to space-filling representation.
Scene 2 : The two bound DNA products now appear. Rotation occurs - note the view down the axis of the coaxial (uncleaved) arms as it passes, also showing that the remaining (cleaved) arms are mutually perpendicular.
Scene 3: One of the two products is recolored so that the four strands are now colored as in the complete junction (Figure 1A).
Scene 4. The strands become reconnected to form an intact junction. The view now zooms into the center of the junction, which is now shown with bonds in stick form. Notice that the reconnected green h strand is now close to the active site, indicated by the magenta metal ion. The strand reconnection was modeled manually – see text for details.

SUPPLEMENTARY TABLE S1

mutant	K_d / nM	rate / s^{-1}
WT	7.7 ± 0.01	0.12 ± 0.01
D38A	6.2 ± 0.05	0.01 ± 0.001
D79A	5.9 ± 0.05	$3.9 \times 10^{-6} \pm 7 \times 10^{-7}$
E120A	6.5 ± 0.10	$0.0012 \pm 7 \times 10^{-6}$
E122A	4.6 ± 0.04	$5.5 \times 10^{-6} \pm 3 \times 10^{-7}$
D141A	10.0 ± 0.01	$4.3 \times 10^{-4} \pm 3 \times 10^{-5}$
D143A	7.1 ± 0.01	0.001 ± 10^{-4}

Table S1. The Cleavage Activity and Binding Affinity of Point Mutants of CtGEN1; Related to Figure 4.
Affinities were measured by separation of free and complexed junction 3 by gel electrophoresis, and data fitted to the Hill equation. There was a spread of Hill coefficients between 5.3 (wild type CtGEN1) and 3.1. The rates of cleavage were measured under single-turnover conditions, and cleavage progress curves fitted to single exponential functions.

MATERIALS AND METHODS

Sample preparation and purification

A synthetic gene encoding *C. thermophilus* GEN1 1-487 with codon optimization for *E. coli* (Invitrogen) (Freeman et al., 2014) was inserted into a pET series plasmid construct that adds a six-histidine tag at the C-terminal end. The plasmid was transformed into *E. coli* BL21(DE3) RIL (Stratagene). A single colony was incubated in 1 l LB culture at 37°C for 8 h, protein expression induced by addition of IPTG at a final concentration of 0.3 mM, and incubated at 18°C for a further 20 h. Cells were harvested by centrifugation and re-suspended in 2x phosphate-buffered saline containing 10 mM imidazole and frozen at -20°C. After thawing at room temperature and sonication, lysed cells were centrifuged at 20,000g. The supernatant containing the recombinant protein was purified in sequential chromatographic steps using a Ni-NTA gravity column (HiTrap™), heparin HP, Superdex 75 10/300 GL gel filtration and Mono S 5/50 GL (GE healthcare) ion exchange. The purified protein was concentrated to ~100 µM in 5 mM Hepes (pH 7.0), 300 mM NaCl and stored on ice until used in crystallization trials. Purified CtGEN1 migrated as a single band on an overloaded polyacrylamide gel in the presence of SDS.

For crystallization, four strands of 30 nt were chemically synthesized using β-cyanoethyl phosphoramidite chemistry (Beaucage and Caruthers, 1981; Sinha et al., 1984). The oligonucleotides were not phosphorylated at their 5'-termini. Fully deprotected oligonucleotides were purified by gel electrophoresis in 15% (w/v) polyacrylamide gels in 90 mM Tris.borate (pH 8.5), 2 mM EDTA (TBE buffer) containing 8 M urea, and recovered by Elutrap (Whatman) electroelution and ethanol precipitation. The four strands were mixed in equimolar ratio in 5 mM Hepes (pH 7.0), 50 mM NaCl at 90°C and slowly cooled to 4°C. The hybridized junction was purified by electrophoresis in 10% polyacrylamide gels in non-denaturing conditions, and recovered by electroelution and ethanol precipitation. The purified junction was dissolved in 5 mM Hepes (pH 7.0), 50 mM NaCl at a concentration of 100 µM.

DNA sequences used for crystallization (all sequences are written 5' to 3') :
b strand : TCCGTCCTAGCAAGGGGCTGCTACCGGAGG
h strand : CCTCCGGTAGCAGCCTGAGCGGTGGTTGGA
r strand : TCCAACCACCGCTCAACTCAACTGCAGTCT
x strand : AGACTGCAGTTGAGTCCTTGCTAGGACGGA

Crystallization

Equal volumes of CtGEN1 protein (either native or selenomethione-substituted) and four-way DNA junction both at 100 μM were mixed together, and $MgCl_2$ added to a final concentration of 4 mM. This was then diluted with an equal volume of 2x crystallization buffer containing 100 mM Hepes (pH 7.5), 20% PEG10000 and 2 μl hanging drops suspended above crystallization buffer at 7°C in sealed wells. Crystals of dimension 100 x 100 x 100 µm grew in 2-3 weeks. They were attached to loops and soaked

in cryo-protectant containing 100 mM Hepes (pH 7.5), 20% PEG10000 and 30% sucrose. An additional 1 mM $MnCl_2$ was added for the Mn^{2+} complexes. The crystals were then dehydrated by vapor diffusion equilibration with saturated KNO_3 at 7°C for 2 h, and stored under liquid nitrogen.

Data collection and structure determination

Data sets for the native CtGEN1 complex in Mg^{2+} or Mn^{2+} ions were acquired at the Diamond synchrotron beamline I24 at 2.5 Å, and at the European Synchrotron Radiation Facility (ESRF), Grenoble at 2.6 Å resolution respectively. A single-wavelength anomalous dispersion (SAD) data set for selenomethonine-containing CtGEN1 complex was acquired at the Diamond synchrotron beamline I03 at 3.15 Å resolution. Data sets were checked by L-test, from which it was concluded that there was no twinning. Initial phases were acquired from the SAD data by locating the 8 selenium atoms with Autosol in the PHENIX suite (Adams et al., 2010). The initial model was generated automatically by PHENIX autobuild wizard, and then applied to the native data sets by molecular replacement using Phaser (McCoy et al., 2007). The model was then adjusted manually using Coot (Emsley et al., 2010), and subjected to several rounds of adjustment and optimization using Coot, REFMAC (Murshudov et al., 2011) and phenix.refine. To avoid any uncertainty arising from the space group determination, the original diffraction data sets were processed in the 21 possible space groups for macromolecules in hP (hexagonal Primitive) lattices. The final refined ctGEN1 protein structure (without DNA) was used as the model to search molecular replacement solutions for each space group using Phaser. We found that only P3121 and P3221 gave the correct solution with a Z-score of 12.7. None of the other space groups gave any solution with Z-score > 5.7. Molecular models are displayed and movies were prepared using PyMOL (DeLano, 2002).

	CtGEN1-SeMet	CtGEN1-Mg^{2+}	CtGEN1-Mn^{2+}
**Data collection**			
Wavelength (Å)	0.9796	0.9750	0.91376
Resolution range (Å)	32.43 - 3.15 (3.23 – 3.15)	69.26 - 2.51 (2.71 - 2.51)	49.29-2.60 (2.74 – 2.60)
Space group	P 3_121	P 3_121	P 3_121
Unit cell (Å, °)	99.08, 99.08,120.67<br>90, 90, 120	98.11, 98.11,119.58<br>90, 90, 120	98.58,98,58,119.62<br>90, 90, 120
Total reflections	241448 (18210)	132486 (27582)	391739 (59675)
Unique reflections	12299 (907)	23324 (4720)	21079(3037)
Multiplicity		5.6 (5.8)	
Anomalous Multiplicity	10.5 (10.5)		9.8 (10.2)
Completeness (%)	99.9 (100.00)	99.70 (100.00)	100 (100)
Mean I/s (I)	18.5 (1.7)	12.8 (1.8)	11.2 (2.0)
Wilson B-factor	100.753	42.53	69.46
R_{merge}	0.108 (1.976)	0.151 (0.764)	0.173 (1.683)
$CC_{1/2}$	0.999 (0.682)	0.991 (0.646)	0.999 (0.701)
Figure of merit	0.355		

**Refinement**		
R-work	0.2232 (0.2934)	0.2085 (0.3007)
R-free	0.2437 (0.3207)	0.2526 (0.3552)
Number of atoms		
macromolecules	7374	3729
ions	2	2
water	29	66
rmsd		
bond lengths (Å)	0.029	0.003
bond angles (°)	0.90	0.75
Average B-factor	66.70	85.80
macromolecules	66.70	85.90
ions	73.70	86.50
water	57.90	76.10
PDB	5CO8	5CNQ

Analysis of cleavage and binding affinity with a four-way DNA junction using point mutants of CtGEN1

Selected putative active site residues of CtGEN1 were individually converted into alanine by PCR of the gene using the Q5 site-directed mutagenesis kit (BioLab) following the manufacturer's instructions. Mutations were verified by DNA sequencing. Mutant CtGEN1 was expressed and purified as a fusion with a six-histidine tag at the C-terminal end and purified by Ni-NTA column (HiTrap™) and heparin HP. Protein concentration was estimated by absorbance at 280 nm using ε^{280} = 50,600 $M^{-1}cm^{-1}$ (per monomer).

Cleavage of junction 3 radioactively [5'-^{32}P]-labeled on the x strand in 10 mM Hepes (pH 7.5), 50 mM NaCl, 5 mM $MgCl_2$, 0.1% BSA, 1 mM DTT, 10 ng/μl calf thymus DNA and 400 nM CtGEN1 at 37ºC was analyzed under single-turnover conditions as described previously (Freeman et al., 2014). Substrate and products were separated by gel electrophoresis under denaturing conditions, and quantified by phosphorimaging using a Fuji BAS 1500 phosphorimager with MacBAS software. The fraction of DNA cleaved at time t (F_t) was fitted by nonlinear regression analysis to the equation:

$$F_t = F_f.(1\text{-exp}(-k_c t)) \qquad \text{[eq. 1]}$$

where F_f is the fraction of DNA cleaved at the end of the reaction and k_c the rate of cleavage. Binding affinity was measured by incubation of radioactively-labeled junction 3 with increasing concentrations of CtGEN1 in 10 mM Hepes (pH 7.5), 50 mM NaCl, 1 mM $CaCl_2$, 0.1% BSA, 1 mM DTT, 10 ng/μl calf thymus DNA for 1 h. at 20°C as described previously (Freeman et al., 2014). Bound and unbound DNA were separated by gel electrophoresis under non-denaturing conditions, and quantified by phosphorimaging using a Fuji BAS 1500 phosphorimager with MacBAS software. Data were analyzed as fraction DNA bound (f_b) versus protein concentration and fitted by non linear regression analysis to the equation :

$$f_b = 1/(1+ (K_d/ P_t)^n) \quad \text{[eq. 2]}$$

where K_d is the dissociation constant, P_t is the total protein monomer concentration. Wild type and mutant CtGEN1 concentrations were 43, 21.4, 19.3, 17.1, 15, 12.8, 10.7, 8.6, 6.4, 4.3 and 2.1 nM and the total concentration of junction 3 was 180 pM.

DNA sequences of strands contained in junction 3 used to study cleavage activity and binding of CtGEN1 mutants, and chemical probing by potassium permanganate :

b-strand : GGCTAAGGGATCCGTCCTAGCAAGGGGCTGCTACCGGAGGCTTACATCGG
h-strand : CCGATGTAAGCCTCCGGTAGCAGCCTGAGCGGTGGTTGGATGTTGACTGC
r-strand : GCAGTCAACATCCAACCACCGCTCAACTCAACTGCAGTCTAGATGGACTG
x-strand : CAGTCCATCTAGACTGCAGTTGAGTCCTTGCTAGGACGGATCCCTTAGCC

Comparative gel electrophoresis

The six possible DNA junctions with the core sequence of junction 3 (Duckett et al., 1988) with two long (40 bp) and two short (14 bp) arms were generated by chemical synthesis and hybridization in appropriate combinations of four strands (Lilley, 2008). Strands were radioactively-[5'-^{32}P]-labeled using T4 polynucleotide kinase (Fermentas) and [γ-^{32}P]ATP (Perkin Elmer). After hybridization by slow cooling, the junction species were purified by electrophoresis in a 6% (29:1) polyacrylamide gel in 90 mM Tris.borate (pH 8.5), 2 mM EDTA, recovered by electroelution and precipitated with ethanol. A fraction of each junction (3 to 10 nM) was incubated with 100 nM CtGEN1 in 10 mM Hepes (pH 7.5), 50 mM NaCl, 0.1% BSA, 1 mM DTT, 5 mM $CaCl_2$ plus 10 ng/ml calf thymus DNA. The six species were then loaded onto a 5% (37:1) polyacrylamide gel in 90 mM Tris.borate (pH 8.5), 2 mM $CaCl_2$. Electrophoresis was performed for 14 h. at 120 V at room temperature, with continuous buffer recirculation at 1 l. h^{-1}. Gels were dried and subjected to phosphorimage analysis using a BAS 1500 phosphorimager with MacBAS software (Fuji).

DNA sequences used in comparative gel electrophoretic analysis :

b strand 80 nt complete :
CGAATTCGACAGGAACCTCGAGGGATCCGTCCTAGCAAGGGGCTGCTACCGGAAGCTTACAGATGTCT
TGCGGGGATCCG
b strand 54 nt 5' end : CGAATTCGACAGGAACCTCGAGGGATCCGTCCTAGCAAGGGGCTGCTACCGGAA
b strand 54 nt 3' end : CCGTCCTAGCAAGGGGCTGCTACCGGAAGCTTACAGATGTCTTGCGGGGATCCG
b strand 14 nt central section : CCGTCCTAGCAAGGGGCTGCTACCGGAA
h strand 80 nt complete :
CGGATCCCCGCAAGACATCTGTAAGCTTCCGGTAGCAGCCTGAGCGGTGGTTGAATTCACAGATGACTG
TTCTGAATTCG
h strand 54 nt 5' end : CGGATCCCCGCAAGACATCTGTAAGCTTCCGGTAGCAGCCTGAGCGGTGGTTGA
h strand 54 nt 3' end : TTCCGGTAGCAGCCTGAGCGGTGGTTGAATTCACAGATGACTGTTCTGAATTCG
h strand 14 nt central section : TTCCGGTAGCAGCCTGAGCGGTGGTTGA

r strand 80 nt complete :

CGAATTCAGAACAGTCATCTGTGAATTCAACCACCGCTCAACTCAACTGCAGTCTAGAACACATGTCCA CAATGGATCCG

r strand 54 nt 5' end : CGAATTCAGAACAGTCATCTGTGAATTCAACCACCGCTCAACTCAACTGCAGTC

r strand 54 nt 3' end : TCAACCACCGCTCAACTCAACTGCAGTCTAGAACACATGTCCACAATGGATCCG

r strand 14 nt central section : TCAACCACCGCTCAACTCAACTGCAGTC

x strand 80 nt complete :

CGGATCCATTGTGGACATGTGTTCTAGACTGCAGTTGAGTCCTTGCTAGGACGGATCCCTCGAGGTTCC TGTCGAATTCG

x strand 54 nt 5' end : CGGATCCATTGTGGACATGTGTTCTAGACTGCAGTTGAGTCCTTGCTAGGACGG

x strand 54 nt 3' end : GACTGCAGTTGAGTCCTTGCTAGGACGGATCCCTCGAGGTTCCTGTCGAATTCG

x strand 14 nt central section : GACTGCAGTTGAGTCCTTGCTAGGACGG

Permanganate probing of the DNA junction

Junction 3 with 25 bp arms radioactively [5'-^{32}P]-labeled on a given strand was subjected to permanganate probing. 10 nM junction with and without 70 nM CtGEN1 was reacted with 1 mM $KMnO_4$ for 2 min in 10 mM Hepes (pH 7.5), 50 mM NaCl, 1 mM $CaCl_2$, 0.1% BSA in a volume of 100 μl at 25ºC. The reaction was terminated by addition of 25 μl of 1 M β-mercaptoethanol, 1M NaCl, 10 mM EDTA, and the DNA precipitated with ethanol. The DNA was recovered by centrifugation, resuspended in 1 M piperidine and incubated at 95ºC for 30 min. After lyophilization the DNA was dissolved in 20 μl water and lyophilization repeated two times. Reactions were performed in the same conditions with the single-stranded h or x strand to generate a thymine-specific sequence marker. The products were separated by electrophoresis in a 10% polyacrylamide gel in TBE containing 8 M urea. The dried gel was exposed to a storage phosphor screen, and the radioactive DNA visualized by phosphorimaging using a Fuji BAS 1500 phosphorimager with MacBAS software.

Fluorescence spectroscopy

Junction 3 with 25 bp arms and its corresponding r-strand resolution cleavage product were prepared with adenine nucleotides substituted by 2-aminopurine at selected single positions. Steady-state fluorescence emission spectra were recorded on an SLM-Aminco 8100 fluorimeter, with excitation at 315 nm in order to minimize the fluorescence of the CtGEN1 protein, which contains seven tryptophan residues. Fluorescence emission was recorded between 330 nm and 460 nm in 1 nm intervals. The excitation and emission monochromators were each set with band passes of 8 nm. Polarization filters were set at the magic angle, and spectra were corrected for lamp fluctuations. All the measurements were performed at 10 °C in a 5 mm × 5 mm cuvettes. Samples contained 200 nM DNA substrate in 20 mM Hepes (pH 7.5), 50 mM NaCl, 1 mM $CaCl_2$, in a total volume of 400 µl. CtGEN1 aliquots were added to final concentrations of 0, 200, 400, 600, 800 nM, 1, 1.2, 1.4 μM for the junction, and 0, 100, 200, 300, 400, 600, 800 nM, 1, 1.2, 1.4 μM for the product DNA. All fluorescence emission spectra and fluorescence intensities were corrected for tryptophan fluorescence and background emission by subtraction of control spectra, using DNA species lacking 2-aminopurine substitution. Spectra were integrated between 370 and 410 nm to calculate binding curves.

2-aminopurine-containing DNA sequences used for fluorescence spectroscopy :

Junction 3 variants were assembled by annealing three unmodified oligonucleotides with a single 2-aminopurine-containing strand. Product variants were assembled by annealing the truncated h and x strands with a 2-aminopurine-containing r strand. In the following sequences, **P** denotes 2-amino-purine and p denotes a 5'-phosphate.

b strand (50 nt) : CCTCGAGGGATCCGTCCTAGCAAGGGGCTGCTACCGGAAGCTTACAGATG

h strand (50 nt) : CATCTGTAAGCTTCCGGTAGCAGCCTGAGCGGTGGTTGAATTCACAGATG

r strand (50 nt) : CATCTGTGAATTCAACCACCGCTCAACTCAACTGCAGTCTAGAACACATG

x strand (50 nt) : CATGTGTTCTAGACTGCAGTTGAGTCCTTGCTAGGACGGATCCCTCGAGG

b strand 2AP at -3 (50 nt) : CCTCGAGGGATCCGTCCTAGCA**P**GGGGCTGCTACCGGAAGCTTACAGATG

h strand 2AP at 3: (50 nt) : CATCTGTAAGCTTCCGGTAGCAGCCTG**P**GCGGTGGTTGAATTCACAGATG

r strand 2AP at -1 (50 nt) : CATCTGTGAATTCAACCACCGCTC**P**ACTCAACTGCAGTCTAGAACACATG

r strand 2AP at +1: (50 nt) : CATCTGTGAATTCAACCACCGCTCA**P**CTCAACTGCAGTCTAGAACACATG

h strand (27-50 nt product) : p-GAGCGGTGGTTGAATTCACAGATG

x strand (1-26 nt product) : CATGTGTTCTAGACTGCAGTTGAGTC

References

Adams, P.D., Afonine, P.V., Bunkoczi, G., Chen, V.B., Davis, I.W., Echols, N., Headd, J.J., Hung, L.W., Kapral, G.J., Grosse-Kunstleve, R.W., *et al*. (2010). PHENIX: a comprehensive Python-based system for macromolecular structure solution. Acta Crystallogr D Biol Crystallog *66*, 213-221.

Beaucage, S.L., and Caruthers, M.H. (1981). Deoxynucleoside phosphoramidites - a new class of key intermediates for deoxypolynucleotide synthesis. Tetrahedron Lett. *22*, 1859-1862.

DeLano, W.L. (2002). The PyMOL Molecular Graphics System (Palo Alto, CA, DeLano Scientific).

Duckett, D.R., Murchie, A.I.H., Diekmann, S., von Kitzing, E., Kemper, B., and Lilley, D.M.J. (1988). The structure of the Holliday junction and its resolution. Cell *55*, 79-89.

Emsley, P., Lohkamp, B., Scott, W.G., and Cowtan, K. (2010). Features and development of Coot. Acta Cryst D *66*, 486-501.

Freeman, A.D.J., Liu, Y., Déclais, A.-C., Gartner, A., and Lilley, D.M.J. (2014). GEN1 from a thermophilic fungus is functionally closely similar to non-eukaryotic junction-resolving enzymes J Molec Biol *426*, 3946-3959.

Lilley, D.M.J. (2008). Analysis of branched nucleic acid structure using comparative gel electrophoresis. Quart Rev Biophys *41*, 1-39.

McCoy, A.J., Grosse-Kunstleve, R.W., Adams, P.D., Winn, M.D., Storoni, L.C., and Read, R.J. (2007). Phaser crystallographic software. Journal of applied crystallography *40*, 658-674.

Sinha, N.D., Biernat, J., McManus, J., and Koster, H. (1984). Polymer support oligonucleotide synthesis XVIII: Use of β-cyanoethyl-N,N-dialkylamino/N-morpholino phosphoramidite of deoxynucleosides for the synthesis of DNA fragments simplifying deprotection and isolation of the final product. Nucleic Acids Res *12*, 4539-4557.

Murshudov, G.N., Skubak, P., Lebedev, A.A., Pannu, N.S., Steiner, R.A., Nicholls, R.A., Winn, M.D., Long, F., and Vagin, A.A. (2011). REFMAC5 for the refinement of macromolecular crystal structures. Acta Cryst D *67*, 355-367.
